# Serotonin Promotes Development and Regeneration of Spinal Motor Neurons in Zebrafish

**DOI:** 10.1016/j.celrep.2015.09.050

**Published:** 2015-10-22

**Authors:** Antón Barreiro-Iglesias, Karolina S. Mysiak, Angela L. Scott, Michell M. Reimer, Yujie Yang (杨宇婕), Catherina G. Becker, Thomas Becker

**Affiliations:** 1 Centre for Neuroregeneration, Edinburgh Medical School, Biomedical Sciences, The Chancellor’s Building, University of Edinburgh, Edinburgh EH16 4SB, UK; 2 Euan MacDonald Centre for MND Research, University of Edinburgh, Edinburgh EH16 4SB, UK; 3 Technische Universität Dresden, DFG-Center for Regenerative Therapies Dresden, Cluster of Excellence at the TU Dresden, Fetscherstraße 105, 01307 Dresden, Germany

## Abstract

In contrast to mammals, zebrafish regenerate spinal motor neurons. During regeneration, developmental signals are re-deployed. Here, we show that, during development, diffuse serotonin promotes spinal motor neuron generation from pMN progenitor cells, leaving interneuron numbers unchanged. Pharmacological manipulations and receptor knockdown indicate that serotonin acts at least in part via 5-HT1A receptors. In adults, serotonin is supplied to the spinal cord mainly (90%) by descending axons from the brain. After a spinal lesion, serotonergic axons degenerate caudal to the lesion but sprout rostral to it. Toxin-mediated ablation of serotonergic axons also rostral to the lesion impaired regeneration of motor neurons only there. Conversely, intraperitoneal serotonin injections doubled numbers of new motor neurons and proliferating pMN-like progenitors caudal to the lesion. Regeneration of spinal-intrinsic serotonergic interneurons was unaltered by these manipulations. Hence, serotonin selectively promotes the development and adult regeneration of motor neurons in zebrafish.

## Highlights

• Serotonin is a remote signal promoting motor neuron regeneration in adult zebrafish
• Serotonin acts on proliferation of pMN-like progenitors
• Serotonin does not affect regeneration of spinal serotonergic neurons
• Exogenous serotonin can compensate for the loss of endogenous serotonergic axons

## Graphical Abstract

**Figure:**
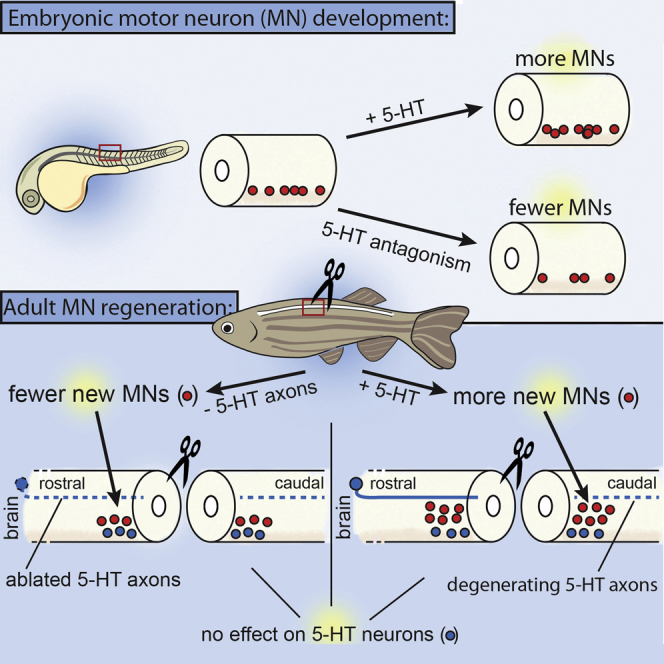

## Teaser

Adult zebrafish, in contrast to mammals, regenerate spinal neurons. Barreiro-Iglesias et al. establish that serotonin from descending axons promotes the regeneration of motor neurons in the lesioned spinal cord but leaves spinal serotonergic neuron regeneration unaffected. Serotonin acts specifically on adult progenitor cells for motor neurons.

## Introduction

In contrast to mammals (Ohori et al., 2006, Su et al., 2014), the CNS of fishes and salamanders regenerates neurons after injury. Ependymo-radial glial cells (ERGs), with a soma forming the ventricular ependyma and radial processes reaching the pial surface, are the likely progenitors (reviewed in Becker and Becker, 2015, Berg et al., 2013, Kizil et al., 2012a). In the spinal cord of adult zebrafish, ERGs are arranged in dorso-ventral domains, similar to progenitors in development (Dessaud et al., 2008), and give rise to distinct cell types after lesion (Kuscha et al., 2012a, Kuscha et al., 2012b, Reimer et al., 2008). For example, motor neurons are regenerated from a ventro-lateral motor neuron progenitor (pMN)-like domain of ERGs, identified by *olig2* expression, after spinal cord transection, whereas serotonergic neurons are regenerated from a more-ventral ERG domain (Kuscha et al., 2012a). Similar ventricular progenitors with the potential to generate neurons exist in the mammalian spinal cord, but in vivo, these cells only give rise to glia (Meletis et al., 2008).

Because of the amazing regenerative capacity of ERGs in zebrafish, it is important to identify the signals that orchestrate neuronal regeneration from these cells. During regeneration of spinal neurons, developmental signals, such as hedgehog (Reimer et al., 2009), notch (Dias et al., 2012), and FGF (Goldshmit et al., 2012) are re-deployed. Dopamine, derived exclusively from descending axons from the diencephalon, is a remote signal promoting motor neuron development and regeneration (Reimer et al., 2013).

Similar to dopamine, serotonin (another monoamine neurotransmitter) is supplied to the adult spinal cord mostly by axons from the brain and may control lesion-induced neurogenesis (Kuscha et al., 2012a, Lillesaar et al., 2009, McLean and Fetcho, 2004). Serotonin promotes developmental (Lauder and Krebs, 1978) and adult neurogenesis in the CNS of mammals (Banasr et al., 2004, Doze and Perez, 2012) and zebrafish (Pérez et al., 2013).

We demonstrate that serotonin promotes spinal motor neuron development and regeneration in zebrafish, supporting the view that motor neuron regeneration from spinal progenitors is specifically regulated by an array of remote and local signals.

## Results

### Serotonin Promotes Motor Neuron Development in Embryonic Zebrafish

In the embryonic spinal cord, most motor neurons are generated between 14 and 48 hr post-fertilization (hpf) (Myers et al., 1986, Reimer et al., 2013). During that time, serotonin is detectable by HPLC in the embryos (Bashammakh et al., 2014).

**Figure 1:**
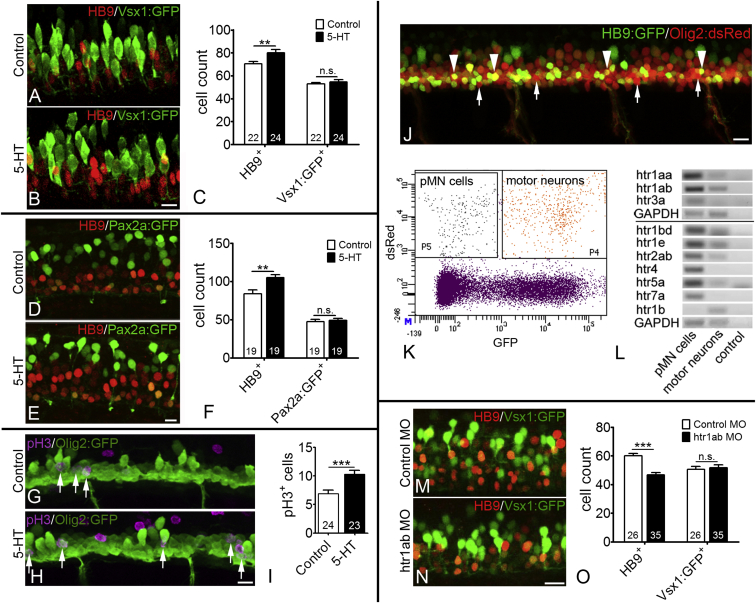
Serotonin Signaling Promotes Embryonic Motor Neuron Generation Lateral views of spinal cords at 33 hpf are shown. (A–F) Serotonin (5-HT) treatment (24–33 hpf) increases the number of HB9 immuno-labeled motor neurons but has no influence on vsx1:GFP (A–C) and pax2a:GFP labeled interneurons (D–F) in the same embryos (Student’s t test in C, ^∗∗^p = 0.0077; in F, ^∗∗^p = 0.002). (G–I) Serotonin treatment increases the number of dividing (pH3^+^) olig2:GFP^+^ pMN progenitor cells (Student’s t test in I; ^∗∗∗^p = 0.0006). (J–L) Lateral view of a double-transgenic olig2:dsRed/HB9:GFP embryo is shown with red only (arrows, pMN progenitors) and double-labeled (arrowheads, motor neurons) cells indicated in the spinal cord (J). A typical FACS profile is shown (K). In RT-PCR, serotonin receptors show enrichment in pMN progenitor cells, compared to motor neurons (L). GAPDH is used for comparison. (M–O) Morpholino knockdown of receptor *htr1ab* reduces the number of HB9^+^ motor neurons but does not influence the number of vsx1:GFP^+^ interneurons in the same embryos (Student’s t test; ^∗∗∗^p < 0.0001). The scale bar in (B) represents 10 μm for (A) and (B), in (E) represents 10 μm for (D) and (E), in (H) represents 15 μm for (G) and (H), and in (N) represents 15 μm for (M) and (N). See also Figure S1.

To directly test the effect of serotonin on spinal neurogenesis, we incubated embryos in serotonin from 24 to 33 hpf. This indicated an increase in the number of HB9^+^ motor neurons, derived from pMN progenitors, of up to 25%. In contrast, the numbers of vsx1:GFP^+^ interneurons (Figures 1A–1C), derived from p2 progenitors, and pax2a:GFP^+^ dorsal interneurons (Figures 1D–1F) remained unchanged in the same embryos. This supports an influence of serotonin on motor neuron generation, but not a generalized effect on spinal neurogenesis.

To determine the source of endogenous serotonin, we used serotonin immunohistochemistry. Serotonin immuno-reactive neurons in the spinal cord and raphe neurons in the brainstem, which later project to the spinal cord, only develop at 48 hpf, and so too late to serve as a cellular source for serotonin (Figures S1A–S1D; McLean and Fetcho, 2004). To determine whether embryonic serotonin synthesis is critical for motor neuron development, we inhibited serotonin synthesis with 50 μM p-chlorophenylalanine (PCPA) from 24 hpf, which effectively abolished serotonin immuno-reactivity at later developmental stages (Figures S1E–S1G). At 33 hpf, the number of HB9^+^ motor neurons and vsx1:GFP^+^ interneurons was unchanged, suggesting that synthesis of serotonin is not required during motor neuron generation (Figure S1H). However, the serotonin re-uptake inhibitor fluvoxamine promoted islet-1:GFP^+^ motor neuron development, probably by raising existing extracellular serotonin levels (Figure S1S). Serotonin may be maternally (Bashammakh et al., 2014) or paternally (Jiménez-Trejo et al., 2012) deposited. Hence, serotonin is present during motor neuron generation, but no cellular source supplying the spinal cord is detectable.

To determine the cellular origin of increased motor neuron numbers, we determined the number of proliferating pMN progenitors. We used olig2:GFP embryos, in which pMN progenitors and motor neurons express GFP, and labeled these with the mitosis marker pH3. Motor neurons are not labeled by pH3, as these are post-mitotic. This indicated a 49% increase in the number of pH3^+^/olig2:GFP^+^ pMN progenitors after serotonin addition (Figures 1G–1I). Prevention of cell death as a reason for increased motor neuron numbers is highly unlikely, because cell death is not observed in the developing ventral spinal cord (Cole and Ross, 2001, Reimer et al., 2013), thus cannot be reduced. Hence, serotonin likely acts, at least in part, by increasing proliferation in the pMN domain.

To detect serotonin receptor expression in pMN progenitors, we used RT-PCR of pMN progenitors (GFP^−^ and dsRed^+^) and motor neurons (GFP^+^ and dsRed^+^), purified by FACS from HB9:GFP/olig2:dsRed double-transgenic zebrafish at 26 hpf (Figures 1J–1L; Reimer et al., 2013). The pMN progenitor fraction contained more *olig2* and *dsRed* mRNA compared to the motor neuron fraction, confirming successful enrichment of progenitor cells (Figure S1I). RT-PCR analysis indicated that, out of 11 serotonin receptors tested, nine (*htr1aa*, *htr1ab*, *htr3a*, *htr1bd*, *htr1e*, *htr2ab*, *htr4*, *htr5a*, and *htr7a*) were more strongly expressed in pMN progenitors compared to motor neurons. *htr1b* was found to be enriched in motor neurons, but not in pMN cells, whereas *htr3b* was not present in either cell type (Figure 1L). This shows that multiple serotonin receptors are enriched in pMN progenitors.

To determine receptors that may mediate the response, we used serotonergic drugs. An agonist of 5-HT1A receptors (8OH-DPAT) increased the number of HB9:GFP^+^ motor neurons by 30% and an antagonist (WAY100635) reduced it by 22% (Figures S1J–S1M). Similarly, 8OH-DPAT also promoted development of islet-1:GFP^+^ motor neurons and WAY100635 inhibited it (Figures S1N–S1R). Moreover, morpholino-mediated knockdown of *htr1ab*, coding for the 5-HT1Ab receptor, reduced numbers of motor neurons, without affecting numbers of vsx1:GFP^+^ neurons (Figures 1M–1O). The absence of an effect on vsx1:GFP matches the observation that serotonin addition does not increase the number of these interneurons and indicates the specific action of the morpholino. This supports that endogenous serotonin acts at least in part through 5-HT1A type receptors to selectively promote motor neuron development.

To test whether serotonin acts by augmenting dopamine signaling, we blocked dopamine-dependent motor neuron generation with the specific Drd4 receptor antagonist L-745870, which inhibits islet-1:GFP^+^ motor neuron development. This cannot be rescued by adding the dopamine agonist pergolide (Reimer et al., 2013). Here, we show that adding serotonin in the presence of L-745870 augments islet-1:GFP^+^ motor neuron development, suggesting that serotonin acts independently of the Drd4a receptor (Figure S1T). However, whereas both pergolide and serotonin increase numbers of HB9^+^ motor neurons, no additive effect was observed (Figure S1U). Hence, serotonin increases motor neuron numbers by increasing proliferation of pMN progenitors in a dopamine-independent way.

### In the Adult Spinal Cord, Serotonergic Axons Contact Spinal Progenitor Cells Rostral to a Lesion

The adult spinal cord receives 90% of its serotonergic innervation from descending axons and the rest from spinal interneurons (Kuscha et al., 2012a). To elucidate a possible influence of serotonin on motor neuron regeneration, we determined the spatial relationship between ERGs and serotonergic axons at 14 days post-lesion (dpl). At that time point, motor neuron regeneration from olig2:GFP^+^ pMN-like ERGs peaks (Reimer et al., 2008) and regeneration of serotonergic neurons are well underway (Kuscha et al., 2012a, Kuscha et al., 2012b).

**Figure 2:**
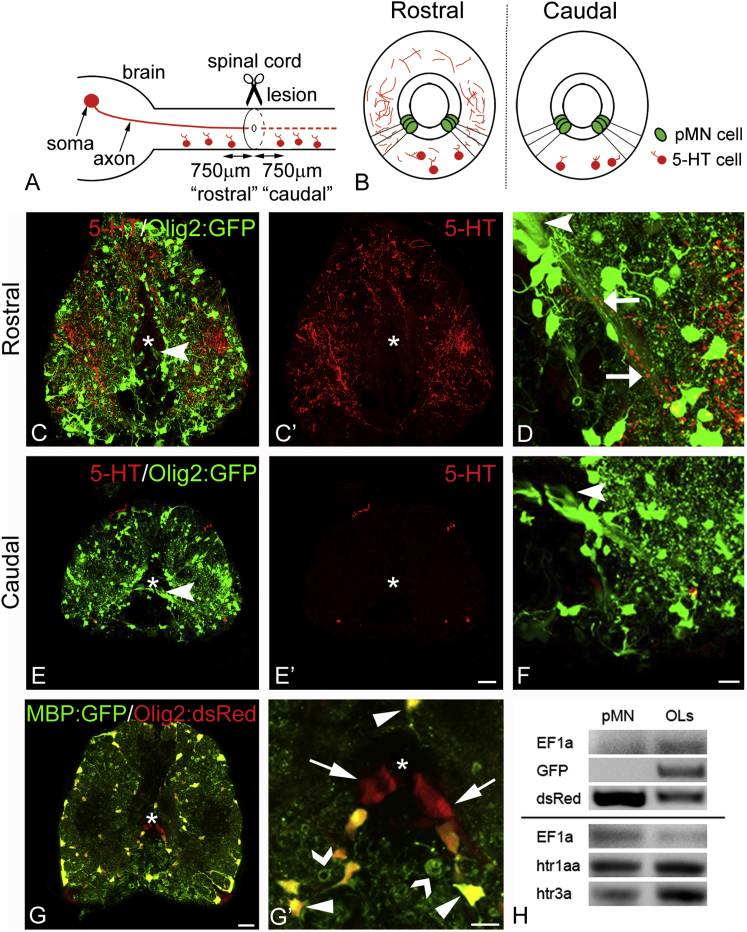
Serotonergic Axons Are Located in Close Proximity to Processes of pMN-like Adult Progenitor Cells Only Rostral to the Lesion Spinal cross-sections are shown (dorsal is up; asterisk indicates central canal). (A and B) Timeline and schematic representations of serotonergic axons in the injured spinal cord in lateral (A) and cross-sectional views (B), indicating the areas of analysis and density of serotonergic axons in relation to pMN-like ERGs. (C–F) Overviews of 5-HT^+^ labeling in olig2:GFP fish rostral (C and C’) and caudal (E and E’) to the lesion are shown. In the ventro-lateral area, close apposition of serotonergic axons (red) with radial processes (green, arrows) of olig2:GFP^+^ pMN-like progenitor cells (arrowheads) are visible rostral (D), but not caudal (F), to the lesion. (G and G’) Olig2:dsRed^+^/mbp:GFP^−^ ERGs (arrows), olig2:dsRed^+^/mbp:GFP^+^ oligodendrocytes (arrowheads), and myelin sheaths (open arrowheads) are indicated (G, overview; G’, detail). (H) PCR after FACS indicates dsRed-only-labeled cells are enriched in the pMN-like ERG fraction, whereas GFP is only amplified in the oligodendrocyte fraction (OLs). EF1alpha is included for comparison. *hrt1aa* and *hrt3a* are expressed in adult pMN-like ERGs. The scale bar in (E’) represents 50 μm for (C), (C’), (E), and (E’); in (F) represents 25 μm for (D) and (F); in (G) represents 25 μm; and in (G’) represents 10 μm. See also Figure S2.

We observed close apposition of some serotonergic axons with the radial processes of the pMN-like ERGs rostral to the lesion (Figures 2A–2D). However, there was no particular association of serotonergic axons with these progenitor cells. Rather, the serotonergic axon distribution appeared diffuse, such that many other ERG types and/or neurons are probably also contacted.

Caudal to the lesion, close apposition of serotonergic axons with radial processes of pMN-like ERGs was rare, because distal severed serotonergic axons had degenerated (Figures 2A, 2B, 2E, 2F, and S2A–S2C; Kuscha et al., 2012a). Axons of local serotonergic neurons are unlikely to replace signaling from descending axons, as their number was very small (7% of the axons present rostral to the lesion; Kuscha et al., 2012a). Hence, serotonin could signal to pMN-like and other ERGs through descending axons rostral, but not caudal to the lesion.

To detect serotonin receptor expression by pMN-like ERGs, we devised a protocol to purify these by FACS. Because the *olig2* regulatory sequences drive expression of fluorescent proteins in pMN-like ERGs and oligodendrocytes in adults, we used a transgenic fish, in which the regulatory sequences of the *myelin basic protein* (*mbp*) gene drive expression of GFP in oligodendrocytes (Almeida et al., 2011) to generate double transgenics with olig2:dsRed. Thus, we were able to remove oligodendrocytes (olig2:dsRed^+^; mbp:GFP^+^) from pMN-like ERGs (olig2:dsRed^+^/mbp:GFP^−^; Figures 2G and 2H) by FACS. RT-PCR of sorted cells indicated expression of *htr1aa* and *hrt3a* (Figure 2H), but not *htr1ab* and *htr1b* (not shown), in both pMN-like ERGs and in oligodendrocytes, known to express serotonin receptors (Fan et al., 2015). This shows that adult pMN-like ERGs express serotonin receptors.

### Ablation of Spinal Serotonergic Axons Inhibits Motor Neuron Regeneration

**Figure 3:**
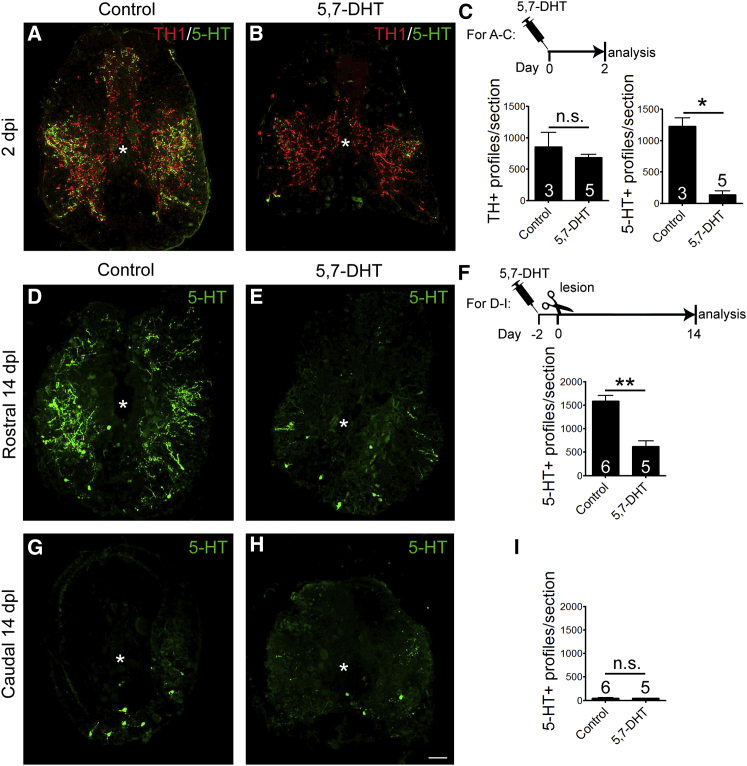
Serotonergic, but Not Dopaminergic, Descending Axons Are Ablated by 5,7-DHT Spinal cross-sections are shown (dorsal is up; asterisk indicates central canal). (A–C) An intraperitoneal injection of 5,7-DHT ablates serotonergic (5-HT), but not TH1^+^, axons within 2 days (Mann-Whitney U-test; ^∗^p = 0.0357). (D–I) At 14 dpl, the number of axons rostral to the lesion is still strongly reduced after prior ablation (D–F), whereas caudal to the lesion, the low axon density is unaltered (G–I; Mann-Whitney U-test; ^∗∗^p = 0.0022). The scale bar in (H) represents 50 μm. See also Figure S3.

To directly test whether serotonergic axons had an influence on the regeneration of neurons in the spinal cord, we established an ablation paradigm using the serotonergic axon specific toxin 5,7-dihydroxytryptamine (5,7-DHT) (Choi et al., 2004). Quantification of serotonergic axon profiles in the spinal cord at 2 days after intraperitoneal injection of 5,7-DHT indicated a loss of 89% of the axons (Figures 3A–3C). Sparsely distributed serotonergic cells in the spinal cord were also significantly reduced in number (control: 30.60 ± 3.19 cells/750 μm, n = 11 animals; 5,7-DHT treated: 14.76 ± 4.19 cells, n = 5; p = 0.0117). In contrast to serotonergic axons, tyrosine hydroxylase 1 (TH1) immuno-reactive, mostly dopaminergic axons in the spinal cord were not affected by the treatment. Directly labeling dopamine also indicated no difference in intensity or number of dopaminergic varicosities (Figure S3). This supports that the 5,7-DHT injections specifically ablate serotonergic axons without reducing dopamine levels in the spinal cord.

Two days after toxin injection, spinal cords were lesioned. At 14 dpl, the number of serotonergic axon profiles in the rostral spinal cord was still 60% lower in the animals injected with 5,7-DHT than in uninjected spinal-lesioned animals (Figures 3D–3F). Caudal to the lesion, there was no significant difference in the very low number of serotonergic axon profiles between control and 5,7-DHT-injected animals, because distal axons degenerated also in controls (Figures 3G–3I). Hence, the density of serotonergic axons was strongly reduced by the toxin only rostral to the lesion for at least 14 dpl.

To determine the number of newly generated motor neurons, we counted strongly HB9 immuno-reactive small cell nuclei. These cells are hardly present in unlesioned spinal cords and massively increase in number around a spinal lesion (Dias et al., 2012). Moreover, using four injections of EdU, we detected co-labeling in 43.52% ± 5.19% of HB9 immuno-reactive small cell nuclei (n = 6; Figures S4A and S4A’). This relatively low labeling rate was expected, as EdU has limited bio-availability (Zeng et al., 2010). Hence, small HB9^+^ nuclei most likely all represent newly generated motor neurons.

**Figure 4:**
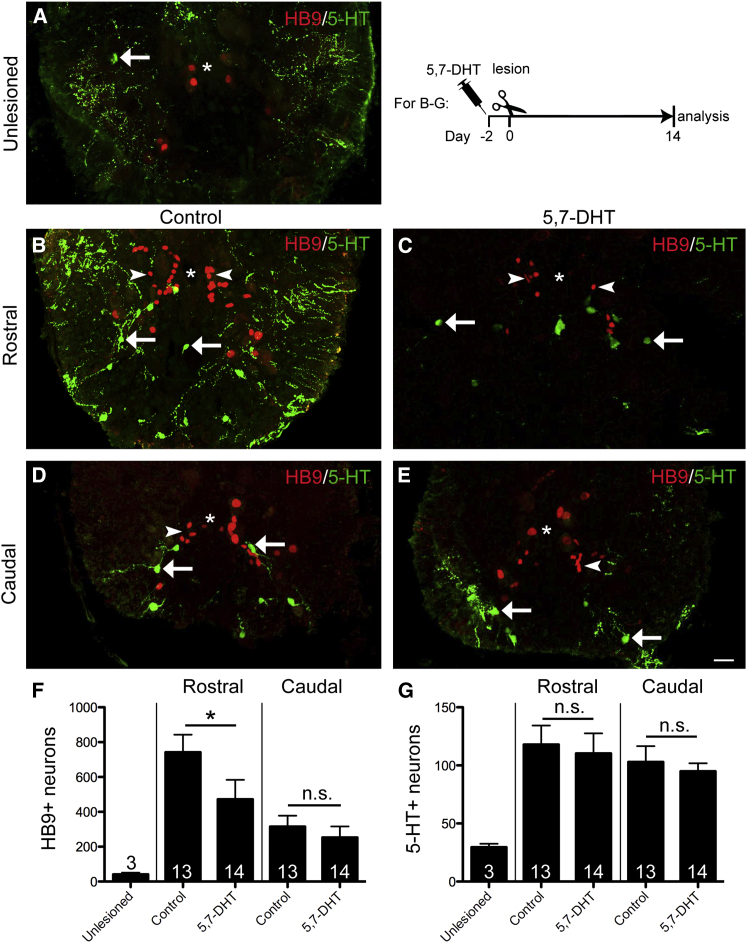
Ablation of Serotonergic Axons Inhibits Regeneration of Motor Neurons, but Not Serotonergic Neurons Spinal cross-sections are shown (dorsal is up; asterisk indicates central canal). (A) In the unlesioned spinal cord, few small HB9^+^ and motor neurons and 5-HT^+^ cells (arrow) are present. (B–G) Rostral to the lesion (see timeline for experimental condition), ablation of serotonergic axons leads to reduced motor neuron (arrowheads) regeneration without influencing regeneration of serotonergic neurons (arrows). Caudal to the lesion, numbers of newly generated motor neurons and serotonergic neurons are unaltered (Mann-Whitney U-test; ^∗^p = 0.0344). The scale bar in (E) represents 25 μm. See also Figure S4.

In control lesioned animals, we found more than twice as many new motor neurons rostral than caudal to the lesion (Figures 4A, 4B, 4D, and 4F; Student’s t test; p = 0.0018). This suggests positive influences of descending axons on motor neuron regeneration that are not present caudal to the lesion due to degeneration of distal axon segments. To exclude positional effects in the spinal cord, we analyzed the equivalent portion of spinal cord with the lesion placed directly caudal instead of rostral to it. This indicated a 67% higher number of newly generated motor neurons (Figures S4B–S4E). This supports that the presence of descending axons, including serotonergic axons, augments motor neuron regeneration.

In 5,7-DHT-treated animals, we found a 36% lower number of new motor neurons than in control lesioned animals rostral to the lesion (Figures 4B, 4C, and 4F). This was unlikely to be due to reduced survival of new motor neurons, as double-labeling of HB9^+^ cells with an antibody to active caspase 3, an indicator of apoptosis (Valencia et al., 2007), was equally low in control and 5,7-DHT-treated animals (Figures S4F–S4I). Caudal to the lesion, as expected from similar low numbers of serotonergic axons in controls and 5,7-DHT-treated animals, no significant difference in the number of newly generated motor neurons was observed (Figures 4D–4F). Moreover, the lack of an effect of 5,7-DHT on motor neuron generation caudal to the lesion indicated the absence of a direct toxic influence of 5,7-DHT on motor neuron regeneration. These data show that serotonergic axons have a positive influence on spinal motor neuron regeneration.

### Serotonergic Axons Do Not Provide a Signal that Affects Lesion-Induced Generation of Spinal Serotonergic Neurons

During unmanipulated regeneration, up to five times the number of serotonergic neurons as in the unlesioned spinal cord are observed at 42 dpl (Kuscha et al., 2012a). In the present experiments, the number of serotonergic cell profiles in the spinal cord close to the lesion was increased to 299% (compared to unlesioned controls) rostral and to 248% caudal to the lesion in controls at 14 dpl. In contrast to motor neuron generation, there was no significant difference in the number of newly generated serotonergic neurons between the rostral and caudal lesioned spinal cord (Figures 4B, 4D, and 4G). This suggested that generation of serotonergic neurons might be independent of descending axons.

Indeed, in 5,7-DHT-treated animals, numbers of serotonergic neurons rose to 273% rostral and to 211% caudal to the lesion, which was not different from controls (Figures 4B–4E and 4G). The fact that a massive reduction of serotonergic axons rostral to the lesion did not affect the increase in the number of serotonergic cell bodies there indicated that generation of serotonergic neurons was not influenced by a serotonin signal. Moreover, equally increased numbers of serotonergic neurons between control and 5,7-DHT-injected animals further supported that 5,7-DHT had no direct non-specific effects on progenitor cells or neuronal differentiation.

### Exogenous Serotonin Augments Regeneration of Motor Neurons

**Figure 5:**
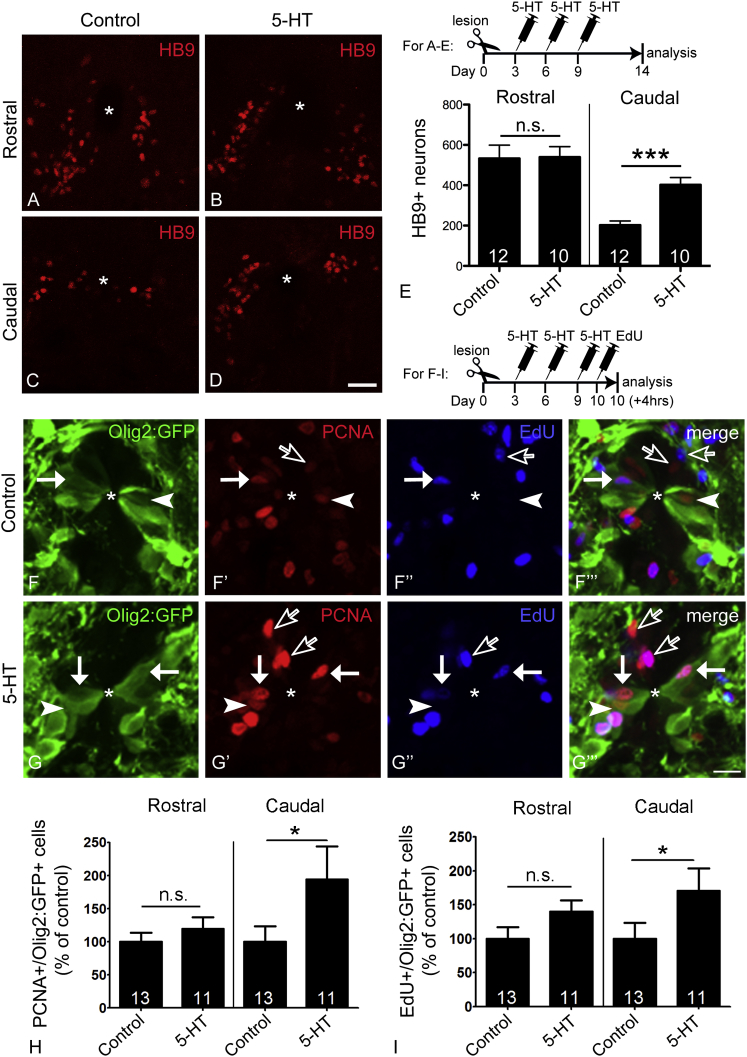
Serotonin Injections Increase the Number of Newly Generated Motor Neurons and Proliferating pMN-like Progenitor Cells Caudal to the Spinal Lesion Cross-sections through the spinal cord are shown; asterisks indicate the central canal, arrowheads indicate PCNA^+^/olig2:GFP^+^ cells, solid arrows indicate EdU^+^/olig2:GFP^+^ cells, and empty arrows indicate PCNA^+^/olig2:GFP^−^ or EdU^+^/olig2:GFP^−^ cells. (A–E) Serotonin injection doubles the number of newly generated motor neurons caudal to the lesion but has no effect rostral to the lesion (see timeline in E for experimental paradigm; Student’s t test; ^∗∗∗^p < 0.0001). (F–I) In the ventricular zone of olig2:GFP transgenic animals, the numbers of PCNA^+^/olig2:GFP^+^ (Mann-Whitney U-test; ^∗^p = 0.0437) and EdU^+^/olig2:GFP^+^ (Mann-Whitney U-test; ^∗^p = 0.0231) pMN-like ERGs are significantly increased only caudal to the lesion (see timeline for experimental paradigm). The scale bar in (D) represents 50 μm for (A)–(D) and in (G’’’) represents 10 μm for (F) and (G). See also Figure S5.

To test whether augmenting the serotonin signal would increase the number of newly generated motor neurons after a spinal lesion, we injected serotonin intraperitoneally at 3, 6, and 9 dpl and analyzed cell numbers at 14 dpl. Indeed, caudal to the lesion, serotonin injections significantly increased the number of small HB9^+^ neurons by 99%, compared to lesioned controls. However, rostral to the lesion, no significant effect of exogenous serotonin was observed, suggesting that endogenous serotonin from sprouting axons rostral to the lesion already had a maximal effect on spinal motor neuron generation (Figures 5A–5E). The number of serotonergic neurons was not significantly altered by serotonin injections (Figures S5A–S5F), consistent with the lack of an effect of ablating serotonergic axons. These findings demonstrate that the augmenting effect of serotonin on the regeneration of motor neurons can be substituted by exogenous serotonin in the spinal cord caudal to a lesion, a region that is deprived of descending serotonergic axons.

To determine whether increasing the serotonin signal in the absence of a spinal lesion was sufficient to induce motor neuron or serotonergic interneuron generation, we injected unlesioned animals with serotonin in the same way as lesioned animals. However, the very low number of small HB9^+^ neurons (control: n = 8, 60.25 ± 10.97; serotonin treated: n = 6, 59.27 ± 10.44; p = 1) as well as that of serotonergic neurons (control: n = 8, 30.99 ± 4.35; serotonin treated: n = 6, 28.84 ± 4.94; p = 0.7546) remained unchanged. This indicates that a lesion signal is needed to make pMN cells competent to react to the serotonin signal.

### Serotonin Increases Lesion-Induced Proliferation in Adult pMNs

Caspase 3 labeling indicated that the effect of serotonin was unlikely on motor neuron survival (see above and Figures S4F–S4I). To determine whether serotonin acted on pMN-like ERGs, we injected lesioned olig2:GFP fish with serotonin at 3, 6, and 9 dpl and analyzed ventricular proliferation at 10 dpl using PCNA and EdU labeling (Figures 5F and 5G). Compared to controls (vehicle-injected lesioned animals), both markers were increased in pMN-like ERGs (PCNA: 94%; EdU: 71%; Figures 5H and 5I). Activation of proliferation in the pMN-like ERG domain caudal to the lesion only is consistent with the rostro-caudal differences in motor neuron generation. Interestingly, outside the pMN-like domain, proliferation was not significantly changed (Figures S5G and S5H). In unlesioned animals, serotonin injections had no effect on proliferative events in the pMN-like domain (PCNA; control: n = 5, 4.49 ± 2.79 cells/750 μm; serotonin treated: n = 4, 9.55 ± 4.13; p = 0.4428). These observations support that pMN-like ERGs are selectively sensitive to the serotonin signal but only become competent to react to it after a lesion.

## Discussion

We demonstrate that serotonin promotes embryonic development and adult regeneration of motor neurons. Serotonin increases proliferation in both embryonic pMN progenitors and adult pMN-like ERGs. Progenitor types in the spinal cord react differently to the serotonin signal, as generation of interneuron populations during development and lesion-induced generation of serotonergic interneurons in adults were not altered by experimental manipulations of serotonin.

Another monoamine signal that stimulates motor neuron development and regeneration is dopamine (Reimer et al., 2013), which is derived from diencephalic neurons in both development and regeneration (Kuscha et al., 2012a, McLean and Fetcho, 2004). In contrast, serotonin appears to be diffusely distributed in embryos, whereas in the adult spinal cord, it is mainly derived from descending brainstem axons (Kuscha et al., 2012a, Lillesaar et al., 2009, McLean and Fetcho, 2004). Another interesting difference is that dopamine attenuates developmental generation of V2 interneurons, whereas serotonin does not influence generation of these interneurons.

Both monoamines likely promote generation of motor neurons independently of each other. In embryos with pharmacologically blocked dopamine signaling, serotonin still promotes motor neuron generation. In lesioned adults, dopamine or serotonin alone promote motor neuron generation caudal to the spinal lesion in the absence of either intact descending dopaminergic or serotonergic axons, as both descending axon types completely degenerate caudal to the lesion (Kuscha et al., 2012a). In addition, ablation of serotonergic axons did not alter spinal dopamine immuno-labeling in the adult spinal cord rostral to the lesion, further supporting that serotonin does not act indirectly through increasing dopamine signaling.

Serotonin likely acts by promoting progenitor proliferation in development and adult regeneration, as addition of serotonin increased the number of proliferating pMN progenitors in embryos. In adults, a doubling of proliferating pMN-like ERGs occurred only caudal to the lesion, which corresponded to a doubling of motor neuron numbers only there. The observation that outside the pMN-like domain no significant increase in proliferation of ventricular cells was elicited suggests that the serotonin signal is relatively selective for pMN-like ERGs. However, we cannot exclude that serotonin acts on neuroblast differentiation in addition to progenitor cell proliferation.

In accordance with the observed increase in progenitor proliferation, pMN progenitors in embryos showed expression of a large number of serotonin receptors. Evidence from treatment with 5-HT1A-specific agonists and antagonists and knockdown of *htr1ab* expression suggests activity of at least this receptor in motor neuron development. Interestingly, adult pMN-like ERGs express *htr1aa*, but not *htr1ab*. This could indicate a switch in receptor usage in adult regeneration. Regeneration-specific gene expression has been observed in zebrafish (Dias et al., 2012, Kizil et al., 2012b). Interestingly, the mammalian homolog *Htr1a* promotes adult neurogenesis in the brain (Banasr et al., 2004). Ependymal cells in spinal-lesioned lampreys also express *htr1a* (Cornide-Petronio et al., 2014). However, several serotonin receptors could be involved in motor neuron regeneration. Of note, adding serotonin in the absence of a lesion is insufficient to induce proliferation or motor neuron generation, indicating that progenitors have to be in a lesion-induced sensitized state to become competent to react to serotonin.

Our present and recent results (Reimer et al., 2013) indicate that the rostral part of the lesioned spinal cord presents a significantly different environment from that in the caudal spinal cord. Regeneration-promoting descending dopaminergic and serotonergic axons present rostral, but not caudal, to a lesion, where these axons degenerate. Local serotonergic neurons in the caudal spinal cord cannot adequately compensate for the lack of descending axons, even though they increase in number after a spinal lesion. That is because their terminals account for only 7% of serotonergic terminals present rostral to the lesion and are rarely close to ERG processes (Figures 2E and 2F). Accordingly, we find twice as many newly generated motor neurons rostral than caudal to the lesion. This difference is not due to intrinsic rostro-caudal differences in the spinal cord, as this phenomenon is independent of the position of the lesion. How descending axons influence post-lesion events in other species needs to be determined.

However, serotonergic innervation from the brain does not influence the lesion-induced generation of serotonergic interneurons, similar to the lack of effect on interneuron types during development. Serotonergic cells are generated in equal numbers rostral and caudal to the lesion despite a much stronger serotonergic innervation rostral to the lesion, and experimental manipulations of the serotonin signal do not influence the number of serotonergic neurons. Thus, progenitors for serotonergic neurons, located ventral to pMNs (Kuscha et al., 2012a), are insensitive to serotonin itself. In contrast, in the hypothalamus of adult zebrafish, serotonin provides positive feedback for the generation of serotonergic neurons (Pérez et al., 2013). Conversely, in the salamander midbrain, dopamine has been found to inhibit regeneration of dopaminergic neurons, thus providing a mechanism by which regeneration is self-limiting (Berg et al., 2011). Our results indicate that serotonin levels in the spinal cord do not control numbers of regenerating serotonergic neurons there. Rather, serotonergic neurons are strongly over-produced after a lesion and only later pruned back in number (Kuscha et al., 2012a). The same is true for motor neurons—only a quarter of the newly generated motor neurons mature, with the rest being eliminated (Reimer et al., 2008). Thus, control of lesion-induced and homeostatic neurogenesis differs between regenerating species, CNS regions, and progenitor type.

In mammals, ependymal cells, which are related to ERGs in fish, proliferate and contribute cells to the glial scar after spinal cord injury, but no new neurons are generated (Meletis et al., 2008). Viral overexpression of transcription factors drives newly generated spinal cells toward neuronal identity (Ohori et al., 2006, Su et al., 2014), but motor neurons are not induced. We show here that, in an adult vertebrate, serotonin significantly increases the number of newly generated motor neurons after a spinal lesion and that exogenous serotonin can replace the endogenous signal.

## Experimental Procedures

### Animals

All fish were kept and bred in our laboratory fish facility according to standard methods (Westerfield, 2000), and all experiments have been approved by the British Home Office. For a list of lines used, see Supplemental Experimental Procedures.

### Drug and Morpholino Treatments

In embryos, drugs were added to the medium from 24 hpf and morpholinos were injected into fertilized eggs, as described (Reimer et al., 2013). Adult fish were anesthetized and injected with substances intraperitoneally at a volume of 25 μl. See Supplemental Experimental Procedures for detail.

### Immunohistochemistry and Quantifications

A list of antibodies and reagents used can be found in the Supplemental Experimental Procedures. Immunohistochemistry on embryo whole mounts and section of the adult spinal cord have been described (Reimer et al., 2013). In embryos, motor neurons and other cell types were counted in confocal image stacks of midthoracic segments, or the percentage of embryos in which spinal islet-1:GFP^+^ motor neurons were present were scored, as described (Reimer et al., 2013). In sections of adult spinal cord, stereological cell counts and fiber counts were performed as described (Kuscha et al., 2012a). All quantifications were done without knowledge of the experimental condition of the specimens. See Supplemental Experimental Procedures for full detail and statistical methods.

### Fluorescence-Activated Cell Sorting and RT-PCR

Motor neurons and pMN cells were isolated from double-transgenic olig2:DsRed/HB9:GFP embryos by FACS of cells from dissociated embryos followed by RT-PCR as described (Reimer et al., 2013). To isolate pMN-like progenitors from the adult spinal cord, we isolated olig2:dsRed^+^/mbp:GFP^−^ cells from double-transgenic animals. See Supplemental Experimental Procedures for detailed protocols and primers used.

### Spinal Cord Lesion

As described previously (Becker et al., 1997), fish were anesthetized by immersion in 0.02% aminobenzoic acid ethyl methyl ester (MS222; Sigma) in PBS for 5 min. A longitudinal incision was made at the side of the fish to expose the vertebral column. The spinal cord was completely transected under visual control, 3.5 mm caudal to the brainstem-spinal cord junction if not indicated differently.

## Author Contributions

A.B.-I., K.S.M., and A.L.S. performed most of the experiments, analyzed data, and critically contributed to manuscript preparation. M.M.R. and Y.Y. contributed experimental data and discussions. C.G.B. and T.B. contributed experiments, oversaw the study, and wrote the manuscript.

## Supplemental Information

Document S1. Supplemental Experimental Procedures and Figures S1–S5

Document S2. Article plus Supplemental Information
