# Supplementary material for: Serotonin Promotes Development and Regeneration of Spinal Motor Neurons in Zebrafish
Source: Cell Rep. 2015 Oct 22;13(5):924–32. doi: 10.1016/j.celrep.2015.09.050 (PMC4635313; doi:10.1016/j.celrep.2015.09.050)
Supplement: Document S1. Supplemental Experimental Procedures and Figures S1–S5 [file mmc1.pdf]

TITLE: Serotonin promotes development and regeneration of spinal motor neurons in zebrafish.

AUTHORS: Antón Barreiro-Iglesias, Karolina S. Mysiak, Angela L. Scott, Michell M. Reimer, Yujie Yang (杨宇婕), Catherina G. Becker, Thomas Becker

SUPPLEMENTAL EXPERIMENTAL PROCEDURES

Animals

We used wild type (*wik*); *Tg(mnx1:GFP)^{ml2}* abbreviated as HB9:GFP (Flanagan-Steet et al., 2005); *Tg(olig2:EGFP),* abbreviated as olig2:GFP (Shin et al., 2003); *Tg(olig2:DsRed2),* abbreviated as olig2:dsRed (Kucenas et al., 2008); *Tg(pax2a:GFP)* abbreviated as pax2a:GFP (Picker et al., 2002); *TgBAC(vsx1:GFP)* abbreviated as vsx1:GFP (Kimura et al., 2008); and *Tg(mbp:EGFP)*, abbreviated as mbp:GFP (Almeida et al., 2011) transgenic lines. Male and female fish were used for the experiments.

Antibodies and reagents

We used mouse anti-HB9 (MNR2, 1:400, Developmental Studies Hybridoma Bank), rabbit anti-serotonin (1:2500, Sigma), mouse anti-PCNA (1:1000, Dako), rabbit anti-pH3 (1:1000, Millipore), chicken anti-GFP (1:500, Abcam), mouse anti-TH1 (1:1000, Millipore) rabbit anti-dopamine (1:750, Dr. H.W.M. Steinbusch, Maastricht University, The Netherlands) and rabbit anti-active caspase 3 (1:500, BD Pharmigen, Cat. No. 559565). Secondary antibodies were donkey anti-mouse Cy3, goat anti-rabbit Dylight-488, donkey anti-chicken Alexa-488 and donkey anti-rabbit Cy3 (all 1:200, Jackson

ImmunoResearch). EdU was detected according to the manufacturer's protocol using Alexa-647 azide as Component B.

Immunohistochemistry and cell quantification in embryos

Immunohistochemistry on embryo whole-mounts has been described (Reimer et al., 2013). Motor neurons and other cell types were counted in confocal image stacks of two or four (pH3 counts only) midthoracic segments as described (Reimer et al., 2013). In some experiments, the percentage of embryos in which spinal islet-1:GFP+ motor neurons were present were scored relative to controls. This was done at least in triplicate with at least 15 embryos per treatment group. This test measures promotion or inhibition of islet-1:GFP+ motor neuron development in a dose-dependent fashion, as confirmed by cell counts and fluorescence intensity measurement (Reimer et al., 2013). For all quantifications, the observer was blinded to the experimental treatments.

Immunohistochemistry and quantifications in adults

Immunohistochemistry on 50 μm vibrating blade microtome sections has been described (Kuscha et al., 2012). Quantification of serotonin, TH1 or dopamine immuno-reactive varicosities in confocal images was done semi-automatically as previously described (Kuscha et al., 2012), except that particles were counted with a particle size from 0 to infinity in Image J (comparing this method with manual counts showed a very good correspondence). Quantification of dopamine immunofluorescence intensity was done with Image J and is given as the mean intensity and the number of

pixels of the highest intensity (255) per spinal cord section (3 sections were analyzed per fish). The observer was blinded to the experimental groups during quantifications.

Cell numbers rostral and caudal to the lesion site were determined in a blinded fashion from stereological counts of confocal images as previously described (Reimer et al., 2013) and schematically depicted in Fig. S1E.

Drug and morpholino treatments in embryos

Drugs (serotonin hydrochloride (5-HT; Sigma H9523), 4-Chloro-DL-phenylalanine (pCPA; Sigma C6506), (±)-8-Hydroxy-2-(dipropylamino)tetralin hydrobromide (8-OH-DPAT; Sigma H8520), fluvoxamine maleate (Sigma F2802), WAY100635 maleate salt (Sigma W108), L-745,870 trihydrochloride (Tocris Bioscience 1002) and pergolide mesylate salt (Sigma P8828) were added to the embryos from 24 hpf at a standard concentration of 10 µM (50 µM for pCPA), as described (Reimer et al., 2013). The *htr1ab* morpholino (5'-ACGTGTCGTTGTTTTCTTCCATGTC -3') and control morpholino (5'-CCTCTTACCTCAGTTACAATTTATA -3') were injected at a concentration of 0.5 mM into fertilized eggs, as described (Reimer et al., 2013).

Intraperitoneal substance applications in adults

Animals were anesthetized and injected intraperitoneally at a volume of 25 µl. 5,7-dihydroxytryptamine (5,7-DHT, Sigma) was dissolved in distilled water and injected at a concentration of 4 mg/ml. Serotonin hydrochloride was dissolved in distilled water and injected at a concentration of 0.2 mg/ml. 5-

ethynyl-2'-deoxyuridine (EdU; InVitrogen, Paisley, UK) was dissolved in 0.3 x Danieau's solution with 15% DMSO to 4.11 mg/ml. Vehicle injections served as controls.

Fluorescence activated cell sorting and RT-PCR in embryos

Motor neurons and pMN cells (25000 and 17000 cells respectively from 200 embryos) were isolated from double transgenic olig2:DsRed/HB9:GFP animals by lysing the embryos and FAC sorting of cells followed by determination of relative receptor expression levels by RT-PCR as described (Reimer et al., 2013). We used the following primers to amplify serotonin receptor sequences: *htr1aa* forward: 5'-GCTTCGAGAACCGAAATGAG-3'; *htr1aa* reverse: 5'-GCTACGATGAAGAAGGGCAG-3'; *htr1ab* forward: 5'-GAGCAAAAACTGGAAAAGCG-3'; *htr1ab* reverse: 5'-GCAGCCAACACAGAATGAAA-3'; *htr1b* forward: 5'-ATCATCGCCATCTCCATCTC-3'; *htr1b* reverse: 5'-GCAGAGGTGAGTCTTTTGCC-3'; *htr1bd* forward: 5'-TGTTCAACAGCTCCGATGAG-3'; *htr1bd* reverse: 5'-AATGGACACCAGGAGGTCTG-3'; *htr1e* forward: 5'-CATATACAGCGCTGCCAAGA-3'; *htr1e* reverse: 5'-GACACAGAAGGCATGCTTGA-3'; *htr2ab* forward: 5'-TTCTGGTCAACACAATCCCA-3'; *htr2ab* reverse: 5'-GCTTTTGGACAGATGGTGGT-3'; *htr3a* forward: 5'-CCAGAGCTGGCTACACACAA-3'; *htr3a* reverse: 5'-GTGAAGCAGCTCCCATTCTC-3'; *htr3b* forward: 5'-AATATCGCTGTCCTCGGATG-3'; *htr3b* reverse: 5'-

TCCACTTCGCTCACTGAATG-3’; *htr4* forward: 5’-CATGGTGAACAAGCCATACG-3’; *htr4* reverse: 5’-CCACCACGTTTGTGATGAAG-3’; *htr5a* forward: 5’-CGACACCTGGAGTACACCCT-3’; *htr5a* reverse: 5’-TCCTCCGAATACGTCTCACC-3’; *htr7a* forward: 5’-GAGAAAGTGCTCATCGGAGG-3’; *htr7a* reverse: 5’-CGGTACAGCACATCACATCC-3’.

For amplification of control sequences we used the following primers: *olig2* forward: 5’-AATGAGCTGCAGAGCATGCG-3’; *olig2* reverse: 5’-TAAGAGTGCCACAACTGGACGG-3’; *dsRed* forward: 5’-AGTTCCAGTACGGCTCCAAG-3’; *dsRed* reverse: 5’-TTGTGGGTCTCGCCCTTCA-3’; *GAPDH* forward: 5’-ACTCCACTCATGGCCGTTAC-3’; *GAPDH* reverse: 5’-TCTTCTGTGTGGCGGTGTAG-3’.

FAC sorting and PCR of adult pMN-like ERGs

For the purification of olig2:dsRed$^+$/MBP:GFP$^-$ cells, we dissociated unlesioned spinal cords from 4 adult olig2:dsRed x MBP:GFP fish, yielding 2041 dsRed$^+$/GFP$^-$ and 941 dsRed$^+$/GFP$^+$ cells. In addition, 2 spinal cords from each WIK, olig2:dsRed, and MBP:GFP lines were used for the calibration of the FAC sorter. The fish were perfused with cold PBS. Spinal cords were dissected and washed twice in calcium-free Ringer buffer. Then the solution was removed and the tissue was minced with a scalpel. The tissue was incubated in EDTA-trypsin (0.25% trypsin and 1mM EDTA in PBS) at room temperature with very gentle agitation for 45 min, followed by

trituration with a pipette. The dissociation was stopped by addition of equal volume of 2 mM CaCl2 with 20% fetal calf serum (FCS) and centrifugation at 3500 g for 5 min. The cells were resuspended in 1ml 2% FCS/2mM EDTA in PBS. The samples were sorted using the BD FACSAria II Flow Cytometer (BD Biosciences, San Jose, California, USA). Live cells were distinguished from debris using DRAQ5 and DAPI staining. The purity of the isolated cells was between 86-97%. Cells were immediately processed for RNA extraction and first-strand cDNA synthesis using Cells Direct One-Step qRT-PCR Kit (Invitrogen). To amplify *EF1* α we used forward: 5'-AGGACATCCGTCGTGGTAAT-3'; reverse: 5'-AGAGATCTGACCAGGGTGGTT-3' primers.

Statistical analyses

Variability of values is always given as SEM. Statistical significance was determined using Student's t-test (for normally distributed data) or Mann-Whitney U-test. We used ANOVA with appropriate post-tests for multiple comparisons.

Literature cited for supplemental experimental procedures

Almeida, R.G., Czopka, T., Ffrench-Constant, C., and Lyons, D.A. (2011). Individual axons regulate the myelinating potential of single oligodendrocytes in vivo. Development *138*, 4443-4450.

Flanagan-Steet, H., Fox, M.A., Meyer, D., and Sanes, J.R. (2005). Neuromuscular synapses can form in vivo by incorporation of initially aneural postsynaptic specializations. Development *132*, 4471-4481.

Kimura, Y., Satou, C., and Higashijima, S. (2008). V2a and V2b neurons are generated by the final divisions of pair-producing progenitors in the zebrafish spinal cord. Development *135*, 3001-3005.

Kucenas, S., Takada, N., Park, H.C., Woodruff, E., Broadie, K., and Appel, B. (2008). CNS-derived glia ensheath peripheral nerves and mediate motor root development. Nat Neurosci *11*, 143-151.

Kuscha, V., Barreiro-Iglesias, A., Becker, C.G., and Becker, T. (2012). Plasticity of tyrosine hydroxylase and serotonergic systems in the regenerating spinal cord of adult zebrafish. J Comp Neurol *520*, 933-951.

Picker, A., Scholpp, S., Bohli, H., Takeda, H., and Brand, M. (2002). A novel positive transcriptional feedback loop in midbrain-hindbrain boundary development is revealed through analysis of the zebrafish pax2.1 promoter in transgenic lines. Development *129*, 3227-3239.

Reimer, M.M., Norris, A., Ohnmacht, J., Patani, R., Zhong, Z., Dias, T.B., Kuscha, V., Scott, A.L., Chen, Y.C., Rozov, S.*, et al.* (2013). Dopamine from the Brain Promotes Spinal Motor Neuron Generation during Development and Adult Regeneration. Dev Cell *25*, 478-491.

Shin, J., Park, H.C., Topczewska, J.M., Mawdsley, D.J., and Appel, B. (2003). Neural cell fate analysis in zebrafish using olig2 BAC transgenics. Methods Cell Sci *25*, 7-14.

SUPPLEMENTAL FIGURES

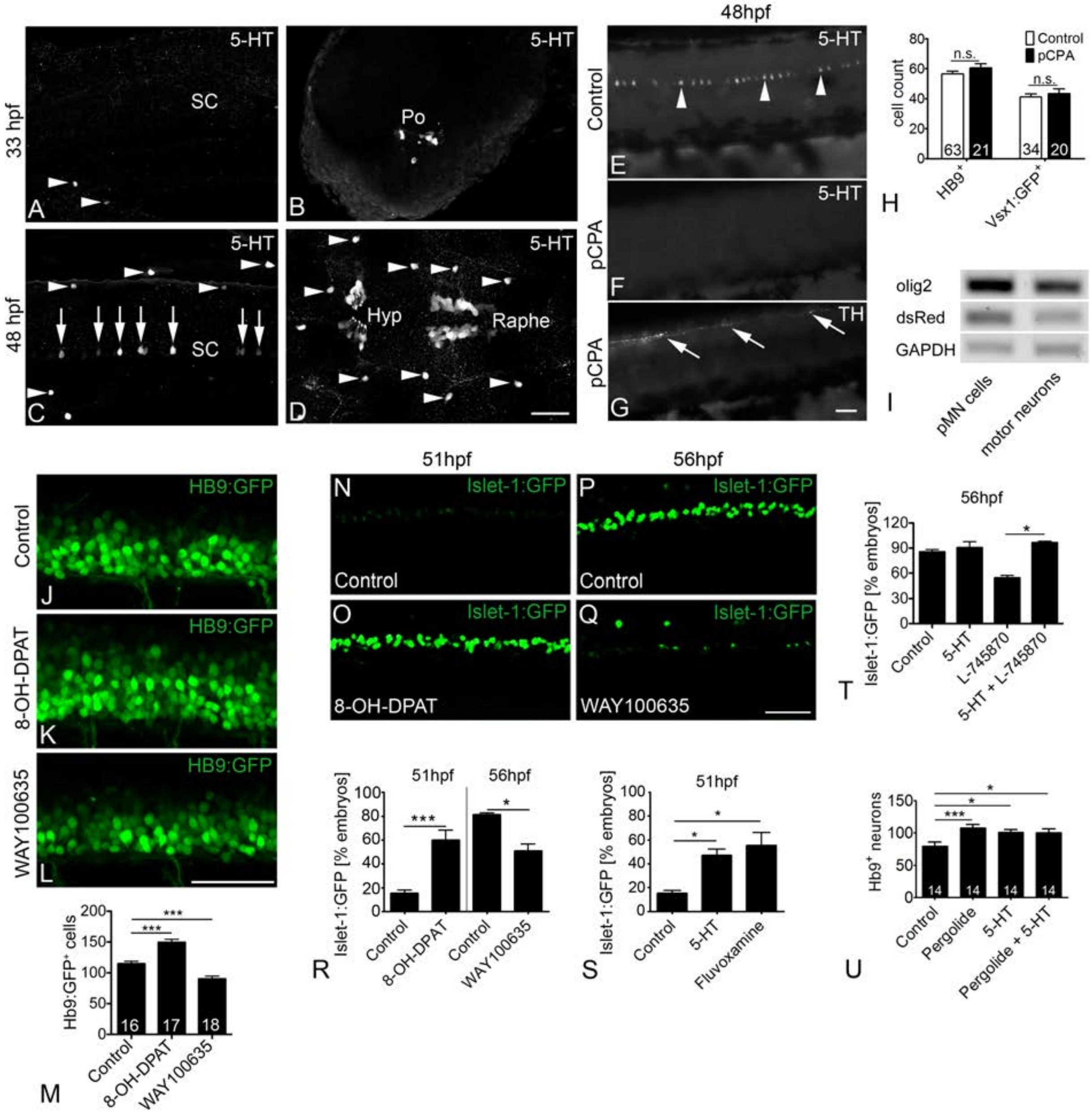

Fig. S1 (related to Fig. 1). Serotonin (5-HT) promotes motor neuron development. **A,C:** Lateral views of the trunk region (left column) indicate that spinal-intrinsic serotonergic cells (arrows) are present at 48, but not 33 hpf. (arrowheads indicate serotonergic cells in the periphery). **B:** A dorsal view of the brain indicates only a few serotonergic cells in the region of the preoptic nucleus (Po) of the diencephalon. **D:** In contrast, large populations of

serotonin-labeled neurons are present in dorsal views in the hypothalamus (Hyp) and raphe region at 48 hpf. **E-G:** Lateral views of the trunk indicate that treatment with pCPA from 24 hpf effectively inhibits serotonin (5-HT) accumulation in spinal neurons (E,F), but does not influence the presence of tyrosine hydroxylase (TH) expressing descending axons (arrows) in the spinal cord (G). **H:** pCPA treatment does not affect numbers of motor neurons or vsx1:GFP+ interneurons at 33 hpf. **I:** Endogenous *olig2* mRNA, as well as *DsRed* mRNA are enriched in FAC sorted progenitor cells, compared to the motor neuron fraction, as shown by RT-PCR in olig2:DsRed and HB9:GFP double transgenic fish. GAPDH is used for comparison. **J-M:** A 5-HT1A agonist (8OH-DPAT) increases and an antagonist (WAY100645) decreases numbers of hb9:GFP+ motor neurons (ANOVA, $P<0.0001$). Lateral views of embryos at trunk level are shown; drug incubation form 24 to 33 hpf. **N-R:** Similarly, 8-OH-DPAT promotes (Student t-test, $P=0.0002$) and WAY100645 inhibits (Mann-Whitney U-test, one-tailed, $P=0.0383$) development of islet-1:GFP+ motor neurons. **S:** Serotonin re-uptake inhibitor fluvoxamine promotes development of islet-1:GFP+ motor neurons, similar to serotonin (ANOVA with Dunn's Multiple Comparison Test, $p < 0.05$). **T:** Serotonin rescues the effect of blocking Drd4 receptors with L-745870 on development of islet-1:GFP+ motor neurons (Mann-Whitney U-test, one-tailed, $p=0.0383$), indicating that serotonin does not act through the Drd4a receptor. **U:** Dopamine agonist pergolide (ANOVA with Bonferroni's Multiple Comparison Test, $p < 0.001$) and serotonin ($p < 0.05$) increase numbers of HB9+ motor neurons, but have no additive effect. Scale bars = 50 μm.

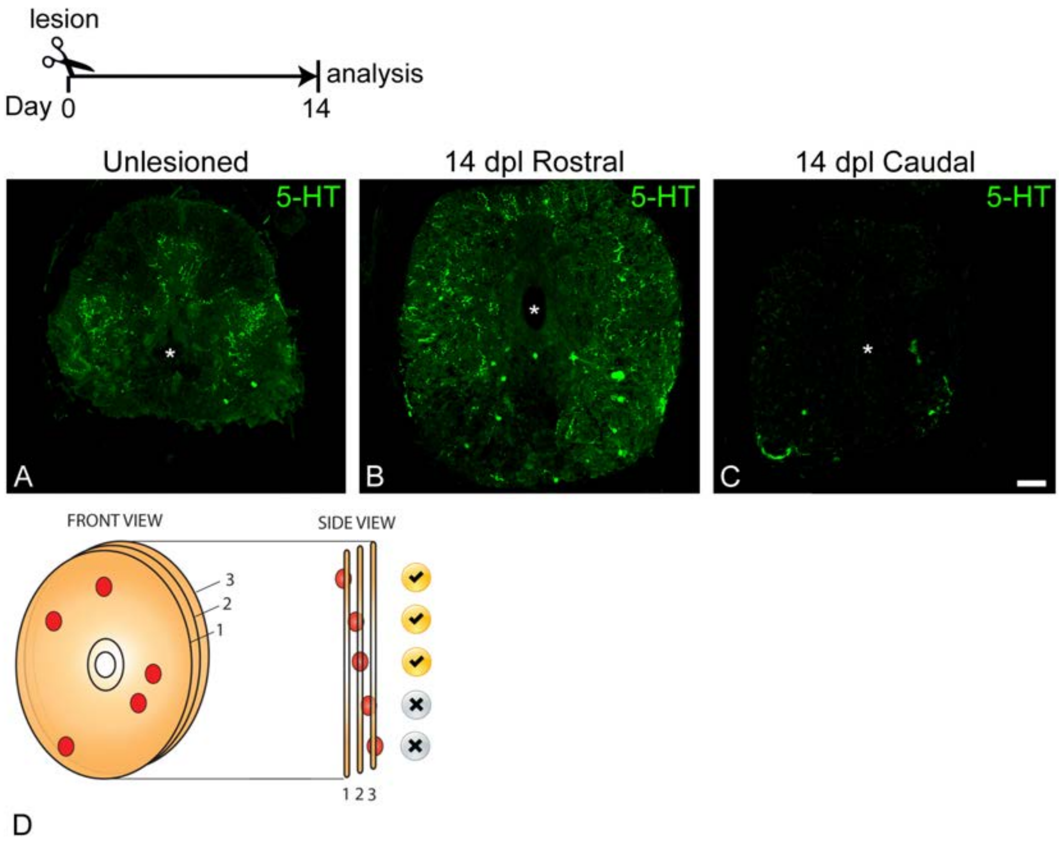

Fig. S2 (related to Fig. 2) Characterization of serotonergic (5-HT) signal and pMN progenitors. **A-C:** Distribution of serotonergic axons in the unlesioned and lesioned spinal cord at 14 days post-lesion is shown in cross sections through the spinal cord; asterisks indicate the central canal. **D:** Schematic representation of cell counts in spinal cord sections. All cells are included in the counts, except for those cells that are visible in the last optical section. Scale bar in C = 50 µm.

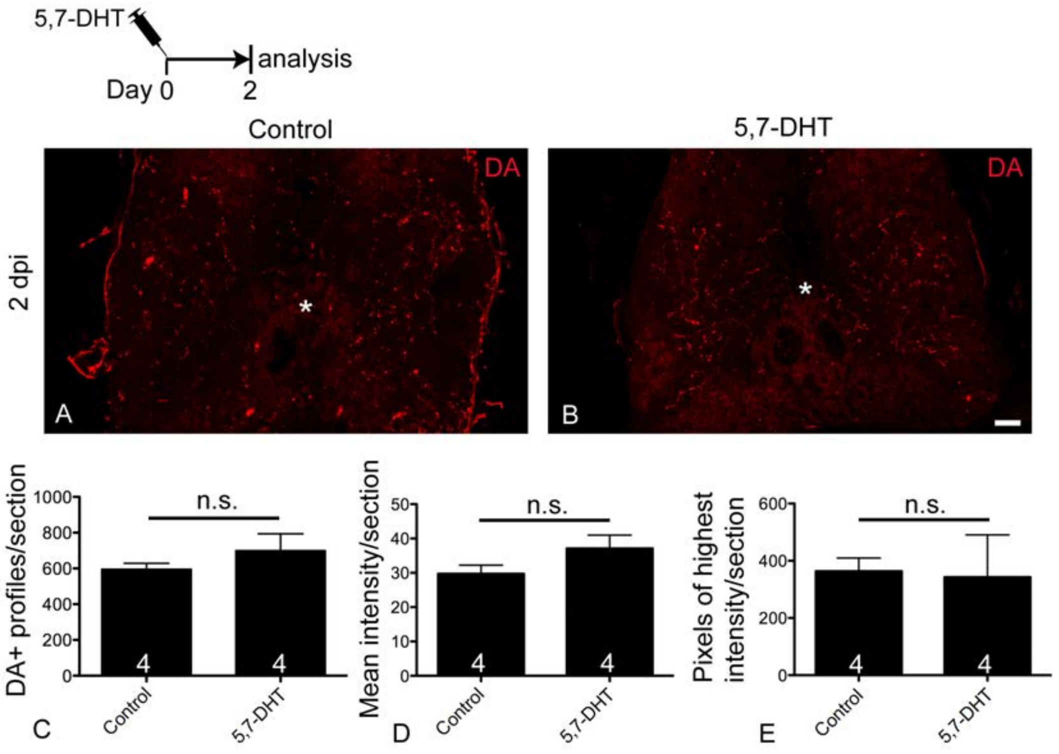

Fig. S3 (related to Fig. 3) Spinal dopamine abundance is not detectably altered after ablation of serotonergic axons with 5,7-DHT. **A,B:** Cross sections through the spinal cord are shown; asterisks indicate the central canal. Immunohistochemistry for dopamine reveals dopaminergic axons in the spinal cord of vehicle-injected and 5,7-DHT injected animals at 2 days post-injection. **C-E:** Number of axonal profiles (Student's t-test, P = 0.3787), as well as mean (P = 0.1875) and peak (P = 0.4724) intensities of dopamine labeling were not detectably different between control and toxin treated animals. Scale bar in B = 50 μm.

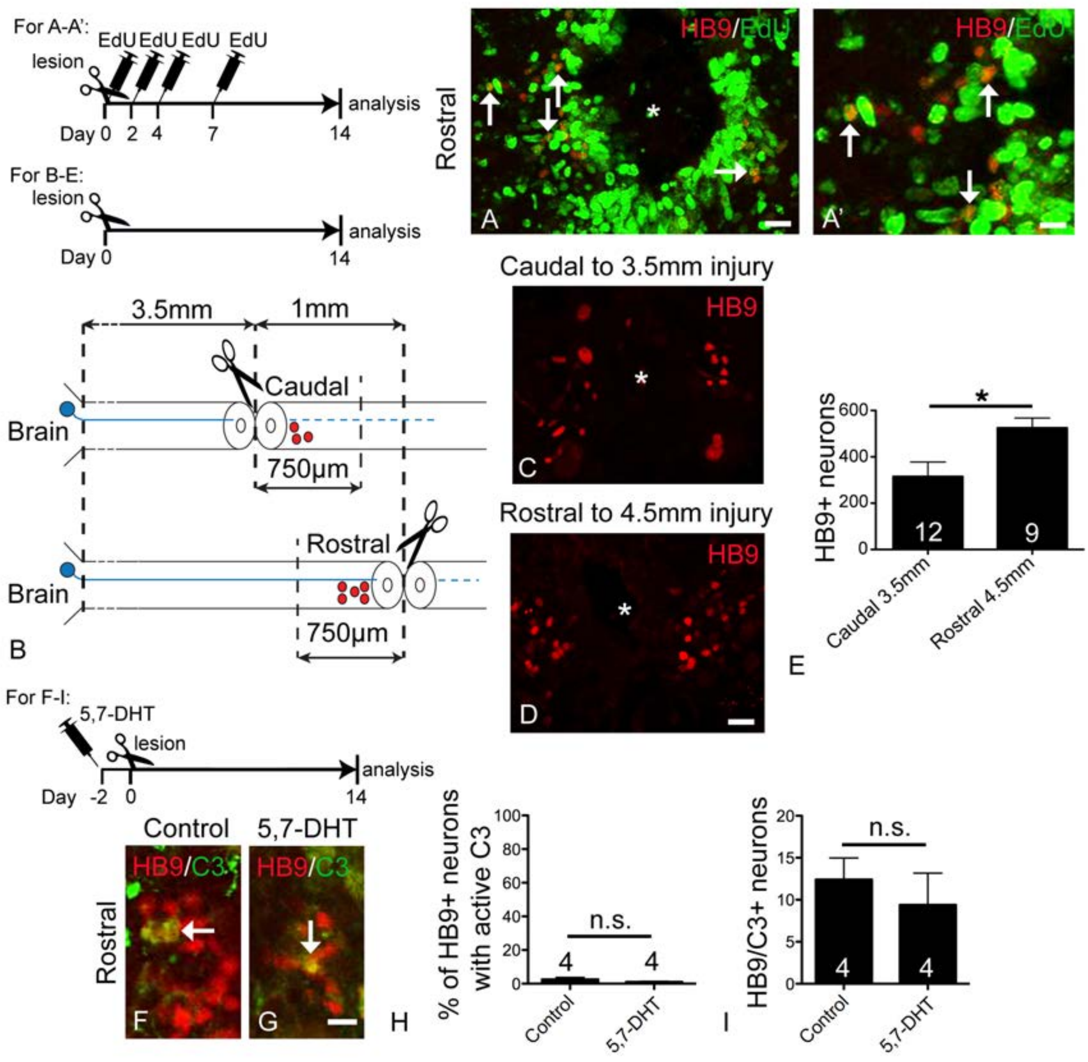

Fig. S4 (related to Fig. 4) Evidence that HB9 immuno-reactive motor neurons are newly generated after a lesion (A, A'), that descending axons influence generation of motor neurons (B-E) and that serotonin signaling does not affect cell death of regenerated motor neurons (F-I). **A,A':** Cross sections through the spinal cord are shown; asterisks indicate the central canal. A' is a higher magnification of the left middle area in A. Multiple EdU injections (see timeline) lead to double labeling of almost half of the newly present HB9$^+$ neurons (arrows) after a lesion. **B-E:** Placing lesions 1 mm apart makes it possible to compare neurogenesis in the same intervening stretch of spinal

cord with either descending axons present (caudal lesion) or absent (rostral lesion) (B). Labeling (C,D) and counts (E) of new $HB9^+$ motor neurons in these equivalent areas up to 750 µm away from the lesion site indicated significantly more new $HB9^+$ motor neurons when the lesion was situated caudal to sampled area (Student's t-test, *P = 0.0184). **F-I:** Double labeling with HB9 and caspase 3 antibodies in control animals and those in which descending serotonergic axons were ablated, did not show increased proportions (Mann-Whitney U-test, P = 0.3429) or numbers (Mann-Whitney U-test, P = 0.6857) of double-labeled cells (arrows) rostral to the lesion site at 14 days post-lesion. Bar in A = 25 µm; in A' = 12.5 µm; in D = 25 µm for C,D; in G = 12.5 µm for F,G.

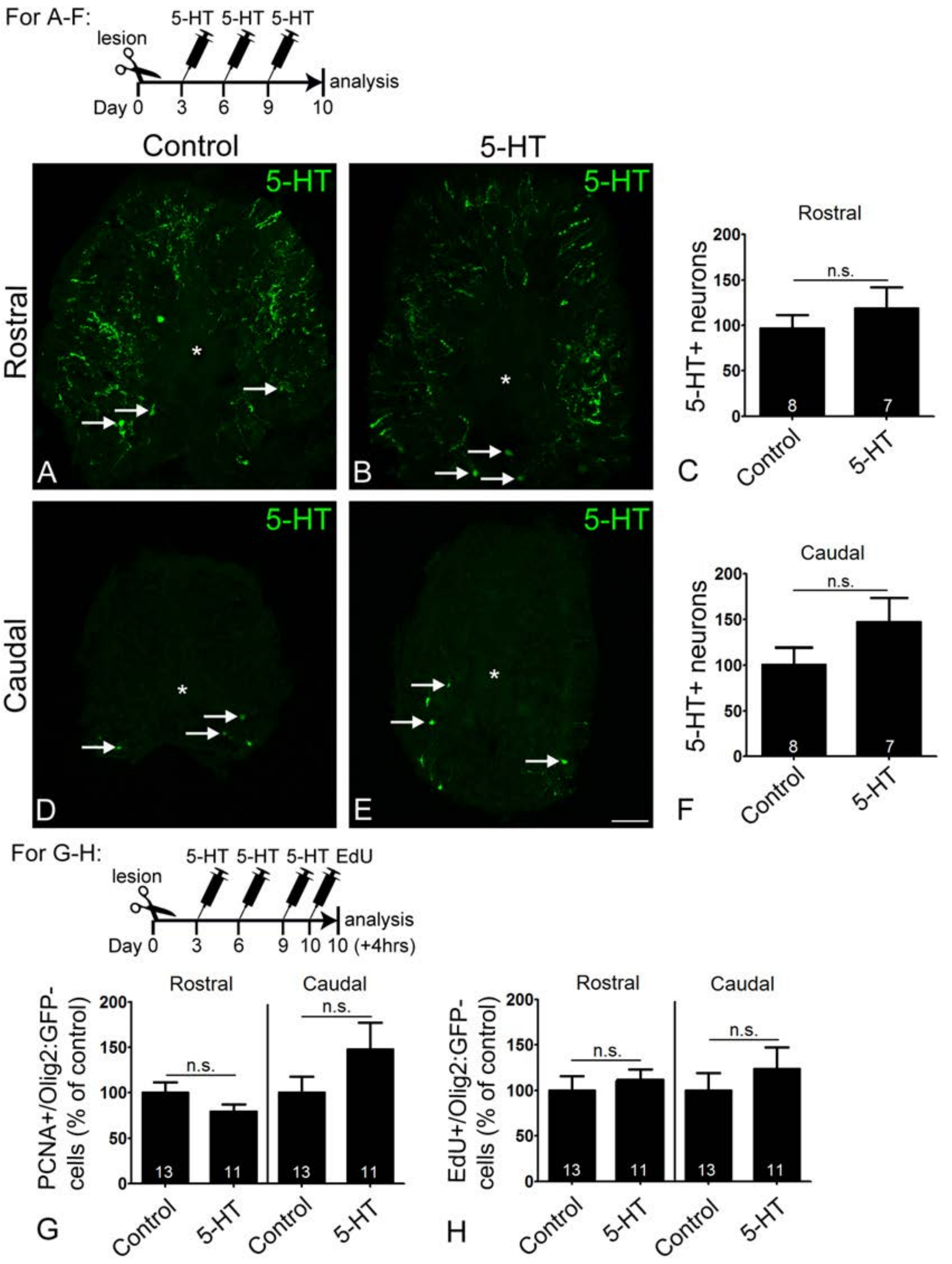

Fig. S5 (related to Fig. 5) Serotonin (5-HT) injections do not increase numbers of serotonergic neurons or proliferation of ventricular cells outside the pMN-like domain. **A-F:** Cross sections through the spinal cord are shown; asterisks indicate the central canal; arrows indicate serotonergic neurons. Serotonin

injections do not significantly increase numbers of serotonergic neurons rostral (Mann-Whitney U-test, P = 0.5358) or caudal (Mann-Whitney U-test, P = 0.2810) to the lesion site. **G,H:** In contrast to the cells in the olig2:GFP^{+} pMN-like domain (compare Fig. 4F-I), the number of PCNA^{+} (Student's t-test, Rostral P = 0.1640, Caudal P = 0.1656) or EdU^{+} (Student's t-test, Rostral P = 0.5525, Caudal P = 0.4323) ventricular cells outside that domain (open arrows in Fig. 4F,G), were not significantly altered by addition of serotonin. Scale bar in E = 50 µm.
