# Supplementary material for: Serotonin Promotes Development and Regeneration of Spinal Motor Neurons in Zebrafish
Source: Cell Rep. 2015 Oct 22;13(5):924–32. doi: 10.1016/j.celrep.2015.09.050 (PMC4635313; doi:10.1016/j.celrep.2015.09.050)
Supplement: Document S2. Article plus Supplemental Information [file mmc2.pdf]

Cell Reports

Report

Serotonin Promotes Development and Regeneration of Spinal Motor Neurons in Zebrafish

Graphical Abstract

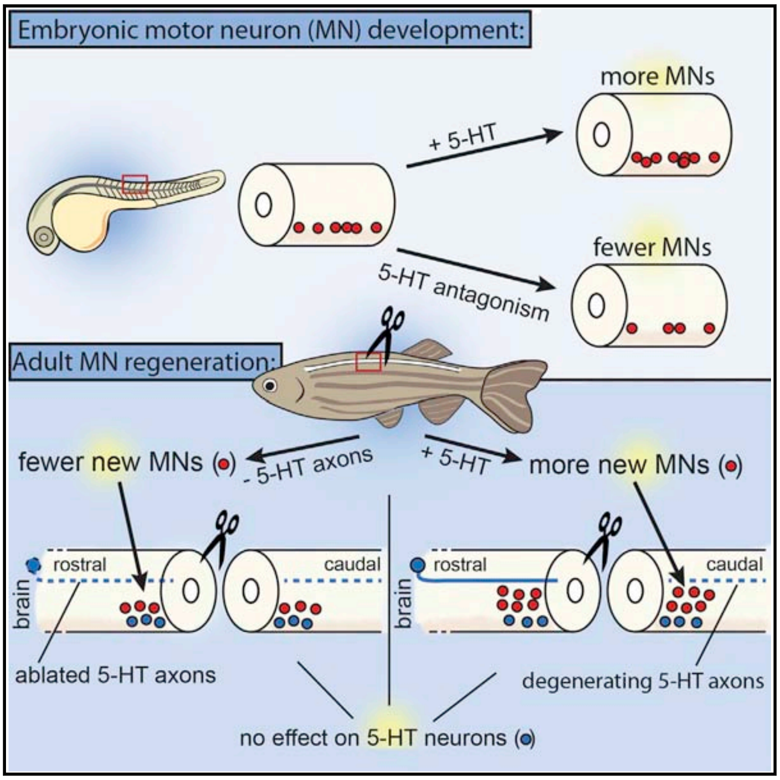

Authors

Antón Barreiro-Iglesias, Karolina S. Mysiak, Angela L. Scott, Michell M. Reimer, Yujie Yang (杨宇婕), Catherina G. Becker, Thomas Becker

Correspondence

catherina.becker@ed.ac.uk (C.G.B.), thomas.becker@ed.ac.uk (T.B.)

In Brief

Adult zebrafish, in contrast to mammals, regenerate spinal neurons. Barreiro-Iglesias et al. establish that serotonin from descending axons promotes the regeneration of motor neurons in the lesioned spinal cord but leaves spinal serotonergic neuron regeneration unaffected. Serotonin acts specifically on adult progenitor cells for motor neurons.

Highlights

- Serotonin is a remote signal promoting motor neuron regeneration in adult zebrafish
- Serotonin acts on proliferation of pMN-like progenitors
- Serotonin does not affect regeneration of spinal serotonergic neurons
- Exogenous serotonin can compensate for the loss of endogenous serotonergic axons

Barreiro-Iglesias et al., 2015, Cell Reports *13*, 924–932
November 3, 2015 ©2015 The Authors
http://dx.doi.org/10.1016/j.celrep.2015.09.050

OPEN ACCESS
CellPress

Cell Reports
Report

Serotonin Promotes Development and Regeneration of Spinal Motor Neurons in Zebrafish

Antón Barreiro-Iglesias,[1,4,6] Karolina S. Mysiak,[1,4] Angela L. Scott,[1,4] Michell M. Reimer,[1,3] Yujie Yang (杨宇婕),[1] Catherina G. Becker,[1,2,5,*] and Thomas Becker[1,2,5,*]
[1]Centre for Neuroregeneration, Edinburgh Medical School, Biomedical Sciences, The Chancellor's Building, University of Edinburgh, Edinburgh EH16 4SB, UK
[2]Euan MacDonald Centre for MND Research, University of Edinburgh, Edinburgh EH16 4SB, UK
[3]Technische Universität Dresden, DFG-Center for Regenerative Therapies Dresden, Cluster of Excellence at the TU Dresden, Fetscherstraße 105, 01307 Dresden, Germany
[4]Co-first author
[5]Co-senior author
[6]Present address: Department of Cell Biology and Ecology, CIBUS, Faculty of Biology, Universidade de Santiago de Compostela, 15782 Santiago de Compostela, Spain
*Correspondence: catherina.becker@ed.ac.uk (C.G.B.), thomas.becker@ed.ac.uk (T.B.)
http://dx.doi.org/10.1016/j.celrep.2015.09.050
This is an open access article under the CC BY license (http://creativecommons.org/licenses/by/4.0/).

SUMMARY

In contrast to mammals, zebrafish regenerate spinal motor neurons. During regeneration, developmental signals are re-deployed. Here, we show that, during development, diffuse serotonin promotes spinal motor neuron generation from pMN progenitor cells, leaving interneuron numbers unchanged. Pharmacological manipulations and receptor knockdown indicate that serotonin acts at least in part via 5-HT1A receptors. In adults, serotonin is supplied to the spinal cord mainly (90%) by descending axons from the brain. After a spinal lesion, serotonergic axons degenerate caudal to the lesion but sprout rostral to it. Toxin-mediated ablation of serotonergic axons also rostral to the lesion impaired regeneration of motor neurons only there. Conversely, intraperitoneal serotonin injections doubled numbers of new motor neurons and proliferating pMN-like progenitors caudal to the lesion. Regeneration of spinal-intrinsic serotonergic interneurons was unaltered by these manipulations. Hence, serotonin selectively promotes the development and adult regeneration of motor neurons in zebrafish.

INTRODUCTION

In contrast to mammals (Ohori et al., 2006; Su et al., 2014), the CNS of fishes and salamanders regenerates neurons after injury. Ependymo-radial glial cells (ERGs), with a soma forming the ventricular ependyma and radial processes reaching the pial surface, are the likely progenitors (reviewed in Becker and Becker, 2015; Berg et al., 2013; Kizil et al., 2012a). In the spinal cord of adult zebrafish, ERGs are arranged in dorso-ventral domains, similar to progenitors in development (Dessaud et al., 2008), and give rise to distinct cell types after lesion (Kuscha et al., 2012a, 2012b; Reimer et al., 2008). For example, motor neurons are regenerated from a ventro-lateral motor neuron progenitor (pMN)-like domain of ERGs, identified by *olig2* expression, after spinal cord transection, whereas serotonergic neurons are regenerated from a more-ventral ERG domain (Kuscha et al., 2012a). Similar ventricular progenitors with the potential to generate neurons exist in the mammalian spinal cord, but in vivo, these cells only give rise to glia (Meletis et al., 2008).

Because of the amazing regenerative capacity of ERGs in zebrafish, it is important to identify the signals that orchestrate neuronal regeneration from these cells. During regeneration of spinal neurons, developmental signals, such as hedgehog (Reimer et al., 2009), notch (Dias et al., 2012), and FGF (Goldshmit et al., 2012) are re-deployed. Dopamine, derived exclusively from descending axons from the diencephalon, is a remote signal promoting motor neuron development and regeneration (Reimer et al., 2013).

Similar to dopamine, serotonin (another monoamine neurotransmitter) is supplied to the adult spinal cord mostly by axons from the brain and may control lesion-induced neurogenesis (Kuscha et al., 2012a; Lillesaar et al., 2009; McLean and Fetcho, 2004). Serotonin promotes developmental (Lauder and Krebs, 1978) and adult neurogenesis in the CNS of mammals (Banasr et al., 2004; Doze and Perez, 2012) and zebrafish (Pérez et al., 2013).

We demonstrate that serotonin promotes spinal motor neuron development and regeneration in zebrafish, supporting the view that motor neuron regeneration from spinal progenitors is specifically regulated by an array of remote and local signals.

RESULTS

Serotonin Promotes Motor Neuron Development in Embryonic Zebrafish

In the embryonic spinal cord, most motor neurons are generated between 14 and 48 hr post-fertilization (hpf) (Myers et al., 1986;

CrossMark

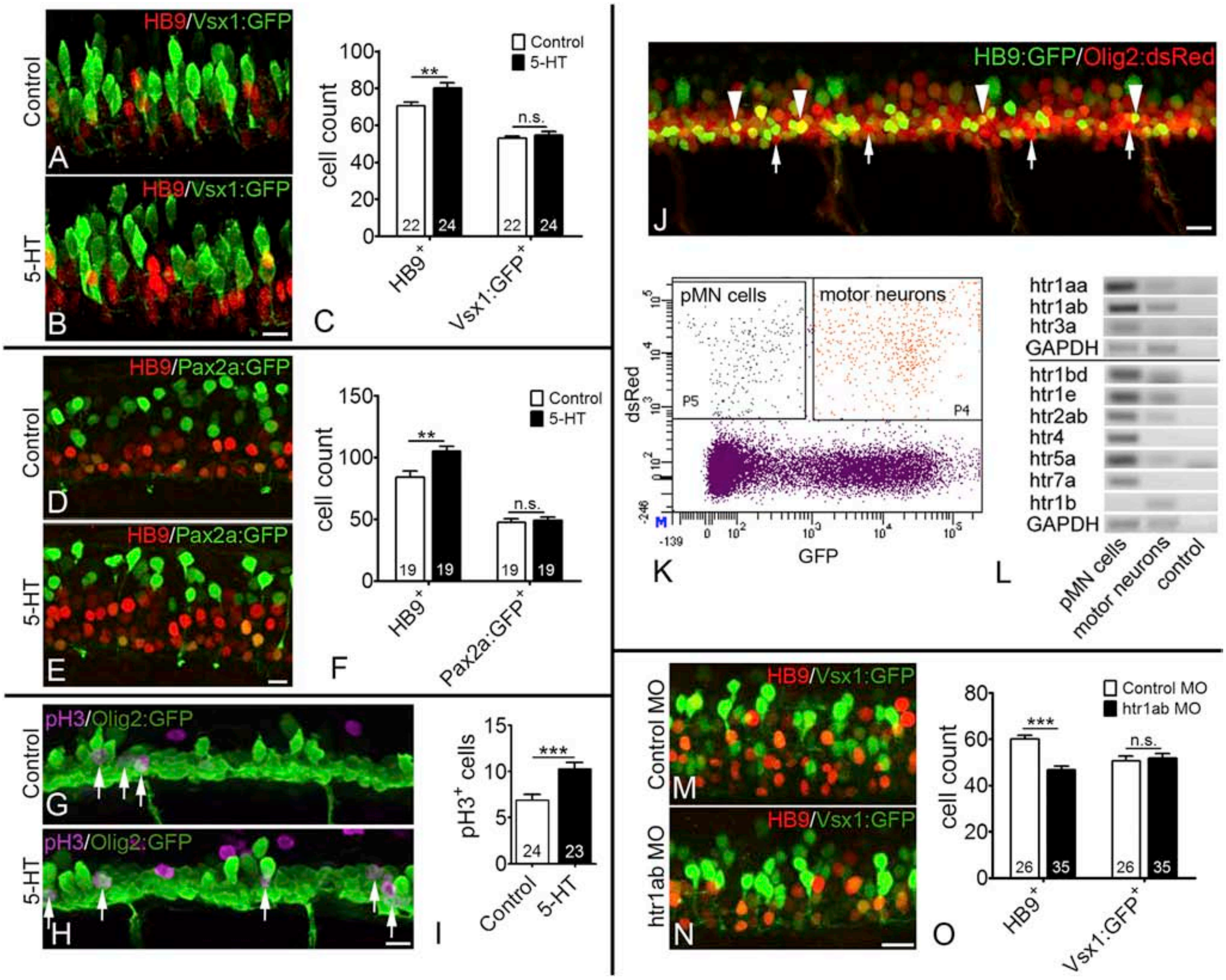

Figure 1. Serotonin Signaling Promotes Embryonic Motor Neuron Generation

Lateral views of spinal cords at 33 hpf are shown.

(A–F) Serotonin (5-HT) treatment (24–33 hpf) increases the number of HB9 immuno-labeled motor neurons but has no influence on vsx1:GFP (A–C) and pax2a:GFP labeled interneurons (D–F) in the same embryos (Student's t test in C, $^{**}p = 0.0077$; in F, $^{**}p = 0.002$).

(G–I) Serotonin treatment increases the number of dividing ($pH3^+$) olig2:GFP$^+$ pMN progenitor cells (Student's t test in I; $^{***}p = 0.0006$).

(J–L) Lateral view of a double-transgenic olig2:dsRed/HB9:GFP embryo is shown with red only (arrows, pMN progenitors) and double-labeled (arrowheads, motor neurons) cells indicated in the spinal cord (J). A typical FACS profile is shown (K). In RT-PCR, serotonin receptors show enrichment in pMN progenitor cells, compared to motor neurons (L). GAPDH is used for comparison.

(M–O) Morpholino knockdown of receptor *htr1ab* reduces the number of $HB9^+$ motor neurons but does not influence the number of vsx1:GFP$^+$ interneurons in the same embryos (Student's t test; $^{***}p < 0.0001$).

The scale bar in (B) represents 10 μm for (A) and (B), in (E) represents 10 μm for (D) and (E), in (H) represents 15 μm for (G) and (H), and in (N) represents 15 μm for (M) and (N). See also Figure S1.

Reimer et al., 2013). During that time, serotonin is detectable by HPLC in the embryos (Bashammakh et al., 2014).

To directly test the effect of serotonin on spinal neurogenesis, we incubated embryos in serotonin from 24 to 33 hpf. This indicated an increase in the number of $HB9^+$ motor neurons, derived from pMN progenitors, of up to 25%. In contrast, the numbers of vsx1:GFP$^+$ interneurons (Figures 1A–1C), derived from p2 progenitors, and pax2a:GFP$^+$ dorsal interneurons (Figures 1D–1F) remained unchanged in the same embryos. This supports an influence of serotonin on motor neuron generation, but not a generalized effect on spinal neurogenesis.

To determine the source of endogenous serotonin, we used serotonin immunohistochemistry. Serotonin immuno-reactive neurons in the spinal cord and raphe neurons in the brainstem, which later project to the spinal cord, only develop at 48 hpf, and so too late to serve as a cellular source for serotonin (Figures S1A–S1D; McLean and Fetcho, 2004). To determine whether embryonic serotonin synthesis is critical for motor neuron

development, we inhibited serotonin synthesis with 50 μM p-chlorophenylalanine (PCPA) from 24 hpf, which effectively abolished serotonin immuno-reactivity at later developmental stages (Figures S1E–S1G). At 33 hpf, the number of $HB9^+$ motor neurons and vsx1:GFP^+ interneurons was unchanged, suggesting that synthesis of serotonin is not required during motor neuron generation (Figure S1H). However, the serotonin re-uptake inhibitor fluvoxamine promoted islet-1:GFP^+ motor neuron development, probably by raising existing extracellular serotonin levels (Figure S1S). Serotonin may be maternally (Bashammakh et al., 2014) or paternally (Jiménez-Trejo et al., 2012) deposited. Hence, serotonin is present during motor neuron generation, but no cellular source supplying the spinal cord is detectable.

To determine the cellular origin of increased motor neuron numbers, we determined the number of proliferating pMN progenitors. We used olig2:GFP embryos, in which pMN progenitors and motor neurons express GFP, and labeled these with the mitosis marker pH3. Motor neurons are not labeled by pH3, as these are post-mitotic. This indicated a 49% increase in the number of $pH3^+$/olig2:GFP^+ pMN progenitors after serotonin addition (Figures 1G–1I). Prevention of cell death as a reason for increased motor neuron numbers is highly unlikely, because cell death is not observed in the developing ventral spinal cord (Cole and Ross, 2001; Reimer et al., 2013), thus cannot be reduced. Hence, serotonin likely acts, at least in part, by increasing proliferation in the pMN domain.

To detect serotonin receptor expression in pMN progenitors, we used RT-PCR of pMN progenitors (GFP^- and $dsRed^+$) and motor neurons (GFP^+ and $dsRed^+$), purified by FACS from HB9:GFP/olig2:dsRed double-transgenic zebrafish at 26 hpf (Figures 1J–1L; Reimer et al., 2013). The pMN progenitor fraction contained more *olig2* and *dsRed* mRNA compared to the motor neuron fraction, confirming successful enrichment of progenitor cells (Figure S1I). RT-PCR analysis indicated that, out of 11 serotonin receptors tested, nine (*htr1aa*, *htr1ab*, *htr3a*, *htr1bd*, *htr1e*, *htr2ab*, *htr4*, *htr5a*, and *htr7a*) were more strongly expressed in pMN progenitors compared to motor neurons. *htr1b* was found to be enriched in motor neurons, but not in pMN cells, whereas *htr3b* was not present in either cell type (Figure 1L). This shows that multiple serotonin receptors are enriched in pMN progenitors.

To determine receptors that may mediate the response, we used serotonergic drugs. An agonist of 5-HT1A receptors (8OH-DPAT) increased the number of HB9:GFP^+ motor neurons by 30% and an antagonist (WAY100635) reduced it by 22% (Figures S1J–S1M). Similarly, 8OH-DPAT also promoted development of islet-1:GFP^+ motor neurons and WAY100635 inhibited it (Figures S1N–S1R). Moreover, morpholino-mediated knockdown of *htr1ab*, coding for the 5-HT1Ab receptor, reduced numbers of motor neurons, without affecting numbers of vsx1:GFP^+ neurons (Figures 1M–1O). The absence of an effect on vsx1:GFP matches the observation that serotonin addition does not increase the number of these interneurons and indicates the specific action of the morpholino. This supports that endogenous serotonin acts at least in part through 5-HT1A type receptors to selectively promote motor neuron development.

To test whether serotonin acts by augmenting dopamine signaling, we blocked dopamine-dependent motor neuron generation with the specific Drd4 receptor antagonist L-745870, which inhibits islet-1:GFP^+ motor neuron development. This cannot be rescued by adding the dopamine agonist pergolide (Reimer et al., 2013). Here, we show that adding serotonin in the presence of L-745870 augments islet-1:GFP^+ motor neuron development, suggesting that serotonin acts independently of the Drd4a receptor (Figure S1T). However, whereas both pergolide and serotonin increase numbers of $HB9^+$ motor neurons, no additive effect was observed (Figure S1U). Hence, serotonin increases motor neuron numbers by increasing proliferation of pMN progenitors in a dopamine-independent way.

In the Adult Spinal Cord, Serotonergic Axons Contact Spinal Progenitor Cells Rostral to a Lesion

The adult spinal cord receives 90% of its serotonergic innervation from descending axons and the rest from spinal interneurons (Kuscha et al., 2012a). To elucidate a possible influence of serotonin on motor neuron regeneration, we determined the spatial relationship between ERGs and serotonergic axons at 14 days post-lesion (dpl). At that time point, motor neuron regeneration from olig2:GFP^+ pMN-like ERGs peaks (Reimer et al., 2008) and regeneration of serotonergic neurons are well underway (Kuscha et al., 2012a, 2012b).

We observed close apposition of some serotonergic axons with the radial processes of the pMN-like ERGs rostral to the lesion (Figures 2A–2D). However, there was no particular association of serotonergic axons with these progenitor cells. Rather, the serotonergic axon distribution appeared diffuse, such that many other ERG types and/or neurons are probably also contacted.

Caudal to the lesion, close apposition of serotonergic axons with radial processes of pMN-like ERGs was rare, because distal severed serotonergic axons had degenerated (Figures 2A, 2B, 2E, 2F, and S2A–S2C; Kuscha et al., 2012a). Axons of local serotonergic neurons are unlikely to replace signaling from descending axons, as their number was very small (7% of the axons present rostral to the lesion; Kuscha et al., 2012a). Hence, serotonin could signal to pMN-like and other ERGs through descending axons rostral, but not caudal to the lesion.

To detect serotonin receptor expression by pMN-like ERGs, we devised a protocol to purify these by FACS. Because the *olig2* regulatory sequences drive expression of fluorescent proteins in pMN-like ERGs and oligodendrocytes in adults, we used a transgenic fish, in which the regulatory sequences of the *myelin basic protein* (*mbp*) gene drive expression of GFP in oligodendrocytes (Almeida et al., 2011) to generate double transgenics with olig2:dsRed. Thus, we were able to remove oligodendrocytes (olig2:$dsRed^+$; mbp:GFP^+) from pMN-like ERGs (olig2:$dsRed^+$/mbp:GFP^-; Figures 2G and 2H) by FACS. RT-PCR of sorted cells indicated expression of *htr1aa* and *hrt3a* (Figure 2H), but not *htr1ab* and *htr1b* (not shown), in both pMN-like ERGs and in oligodendrocytes, known to express serotonin receptors (Fan et al., 2015). This shows that adult pMN-like ERGs express serotonin receptors.

Ablation of Spinal Serotonergic Axons Inhibits Motor Neuron Regeneration

To directly test whether serotonergic axons had an influence on the regeneration of neurons in the spinal cord, we

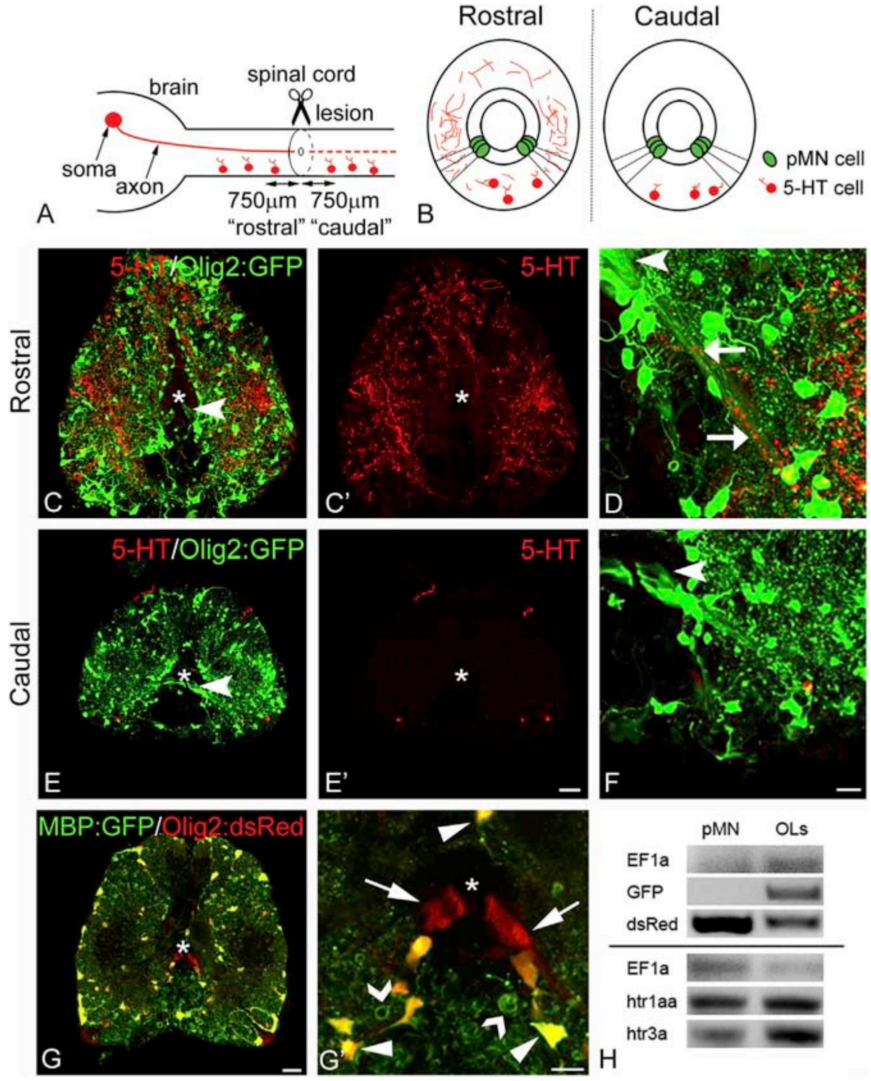

Figure 2. Serotonergic Axons Are Located in Close Proximity to Processes of pMN-like Adult Progenitor Cells Only Rostral to the Lesion

Spinal cross-sections are shown (dorsal is up; asterisk indicates central canal).

(A and B) Timeline and schematic representations of serotonergic axons in the injured spinal cord in lateral (A) and cross-sectional views (B), indicating the areas of analysis and density of serotonergic axons in relation to pMN-like ERGs.

(C–F) Overviews of 5-HT$^+$ labeling in olig2:GFP fish rostral (C and C') and caudal (E and E') to the lesion are shown. In the ventro-lateral area, close apposition of serotonergic axons (red) with radial processes (green, arrows) of olig2:GFP$^+$ pMN-like progenitor cells (arrowheads) are visible rostral (D), but not caudal (F), to the lesion.

(G and G') Olig2:dsRed$^+$/mbp:GFP$^-$ ERGs (arrows), olig2:dsRed$^+$/mbp:GFP$^+$ oligodendrocytes (arrowheads), and myelin sheaths (open arrowheads) are indicated (G, overview; G', detail).

(H) PCR after FACS indicates dsRed-only-labeled cells are enriched in the pMN-like ERG fraction, whereas GFP is only amplified in the oligodendrocyte fraction (OLs). EF1alpha is included for comparison. *hrt1aa* and *hrt3a* are expressed in adult pMN-like ERGs.

The scale bar in (E') represents 50 μm for (C), (C'), (E), and (E'); in (F) represents 25 μm for (D) and (F); in (G) represents 25 μm; and in (G') represents 10 μm. See also Figure S2.

established an ablation paradigm using the serotonergic axon specific toxin 5,7-dihydroxytryptamine (5,7-DHT) (Choi et al., 2004). Quantification of serotonergic axon profiles in the spinal cord at 2 days after intraperitoneal injection of 5,7-DHT indicated a loss of 89% of the axons (Figures 3A–3C). Sparsely distributed serotonergic cells in the spinal cord were also significantly reduced in number (control: 30.60 ± 3.19 cells/750 μm, n = 11 animals; 5,7-DHT treated: 14.76 ± 4.19 cells, n = 5; p = 0.0117). In contrast to serotonergic axons, tyrosine hydroxylase 1 (TH1) immuno-reactive, mostly dopaminergic axons in the spinal cord were not affected by the treatment. Directly labeling dopamine also indicated no difference in intensity or number of dopaminergic varicosities (Figure S3). This supports that the 5,7-DHT injections specifically ablate serotonergic axons without reducing dopamine levels in the spinal cord.

Two days after toxin injection, spinal cords were lesioned. At 14 dpl, the number of serotonergic axon profiles in the rostral spinal cord was still 60% lower in the animals injected with 5,7-DHT than in uninjected spinal-lesioned animals (Figures 3D–3F). Caudal to the lesion, there was no significant difference in the very low number of serotonergic axon profiles between control and 5,7-DHT-injected animals, because distal axons degenerated also in controls (Figures 3G–3I). Hence, the density of serotonergic axons was strongly reduced by the toxin only rostral to the lesion for at least 14 dpl.

To determine the number of newly generated motor neurons, we counted strongly HB9 immuno-reactive small cell nuclei. These cells are hardly present in unlesioned spinal cords and massively increase in number around a spinal lesion (Dias et al., 2012). Moreover, using four injections of EdU, we detected co-labeling in 43.52% ± 5.19% of HB9 immuno-reactive small cell nuclei (n = 6; Figures S4A and S4A'). This relatively low labeling rate was expected, as EdU has limited bio-availability (Zeng et al., 2010). Hence, small HB9$^+$ nuclei most likely all represent newly generated motor neurons.

In control lesioned animals, we found more than twice as many new motor neurons rostral than caudal to the lesion (Figures 4A, 4B, 4D, and 4F; Student's t test; p = 0.0018). This suggests positive influences of descending axons on motor neuron regeneration that are not present caudal to the lesion due to degeneration of distal axon segments. To exclude positional effects in the spinal cord, we analyzed the equivalent portion of spinal cord

OPEN
ACCESS
CellPress

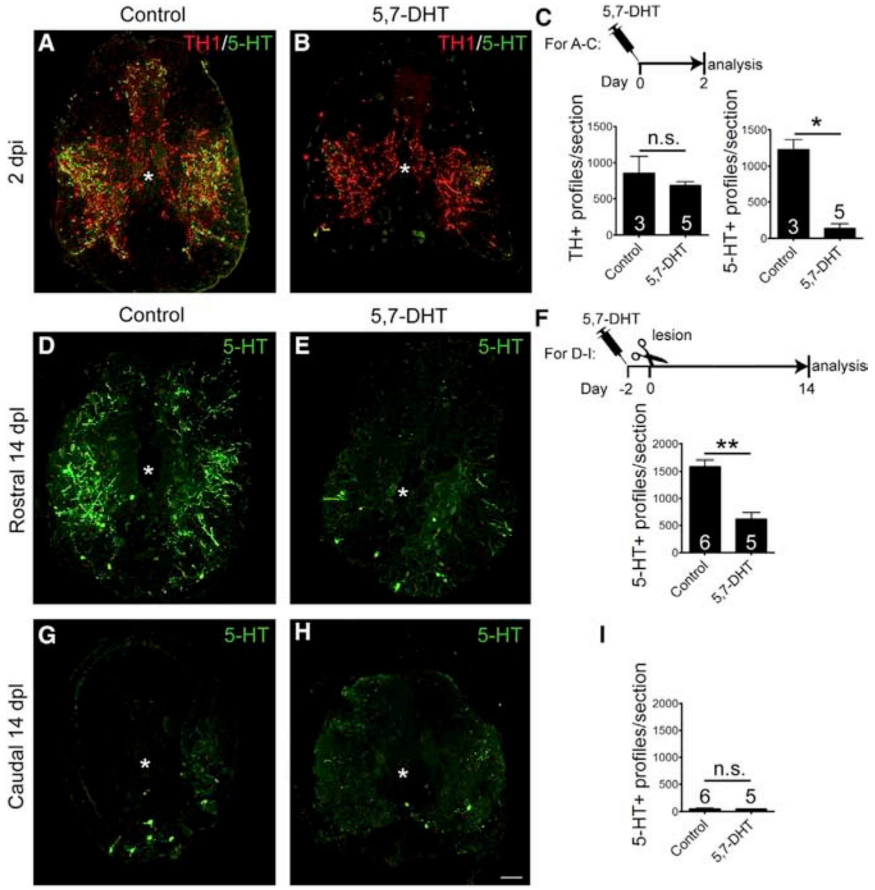

Figure 3. Serotonergic, but Not Dopaminergic, Descending Axons Are Ablated by 5,7-DHT

Spinal cross-sections are shown (dorsal is up; asterisk indicates central canal).

(A–C) An intraperitoneal injection of 5,7-DHT ablates serotonergic (5-HT), but not $TH1^+$, axons within 2 days (Mann-Whitney U-test; $^*p = 0.0357$).

(D–I) At 14 dpl, the number of axons rostral to the lesion is still strongly reduced after prior ablation (D–F), whereas caudal to the lesion, the low axon density is unaltered (G–I; Mann-Whitney U-test; $^{**}p = 0.0022$). The scale bar in (H) represents 50 μm.

See also Figure S3.

with the lesion placed directly caudal instead of rostral to it. This indicated a 67% higher number of newly generated motor neurons (Figures S4B–S4E). This supports that the presence of descending axons, including serotonergic axons, augments motor neuron regeneration.

In 5,7-DHT-treated animals, we found a 36% lower number of new motor neurons than in control lesioned animals rostral to the lesion (Figures 4B, 4C, and 4F). This was unlikely to be due to reduced survival of new motor neurons, as double-labeling of $HB9^+$ cells with an antibody to active caspase 3, an indicator of apoptosis (Valencia et al., 2007), was equally low in control and 5,7-DHT-treated animals (Figures S4F–S4I). Caudal to the lesion, as expected from similar low numbers of serotonergic axons in controls and 5,7-DHT-treated animals, no significant difference in the number of newly generated motor neurons was observed (Figures 4D–4F). Moreover, the lack of an effect of 5,7-DHT on motor neuron generation caudal to the lesion indicated the absence of a direct toxic influence of 5,7-DHT on motor neuron regeneration. These data show that serotonergic axons have a positive influence on spinal motor neuron regeneration.

Serotonergic Axons Do Not Provide a Signal that Affects Lesion-Induced Generation of Spinal Serotonergic Neurons

During unmanipulated regeneration, up to five times the number of serotonergic neurons as in the unlesioned spinal cord are observed at 42 dpl (Kuscha et al., 2012a). In the present experiments, the number of serotonergic cell profiles in the spinal cord close to the lesion was increased to 299% (compared to unlesioned controls) rostral and to 248% caudal to the lesion in controls at 14 dpl. In contrast to motor neuron generation, there was no significant difference in the number of newly generated serotonergic neurons between the rostral and caudal lesioned spinal cord (Figures 4B, 4D, and 4G). This suggested that generation of serotonergic neurons might be independent of descending axons.

Indeed, in 5,7-DHT-treated animals, numbers of serotonergic neurons rose to 273% rostral and to 211% caudal to the lesion, which was not different from controls (Figures 4B–4E and 4G). The fact that a massive reduction of serotonergic axons rostral to the lesion did not affect the increase in the number of serotonergic cell bodies there indicated that generation of serotonergic neurons was not influenced by a serotonin signal. Moreover, equally increased numbers of serotonergic neurons between control and 5,7-DHT-injected animals further supported that 5,7-DHT had no direct non-specific effects on progenitor cells or neuronal differentiation.

Exogenous Serotonin Augments Regeneration of Motor Neurons

To test whether augmenting the serotonin signal would increase the number of newly generated motor neurons after a spinal lesion, we injected serotonin intraperitoneally at 3, 6, and 9 dpl and analyzed cell numbers at 14 dpl. Indeed, caudal to the lesion, serotonin injections significantly increased the number of small $HB9^+$ neurons by 99%, compared to lesioned controls. However, rostral to the lesion, no significant effect of exogenous serotonin was observed, suggesting that endogenous serotonin from sprouting axons rostral to the lesion already had a maximal effect on spinal motor neuron generation (Figures 5A–5E). The number of serotonergic neurons was not significantly altered by serotonin injections (Figures S5A–S5F), consistent with the lack of an effect of ablating serotonergic axons. These findings demonstrate that the augmenting effect of serotonin on the

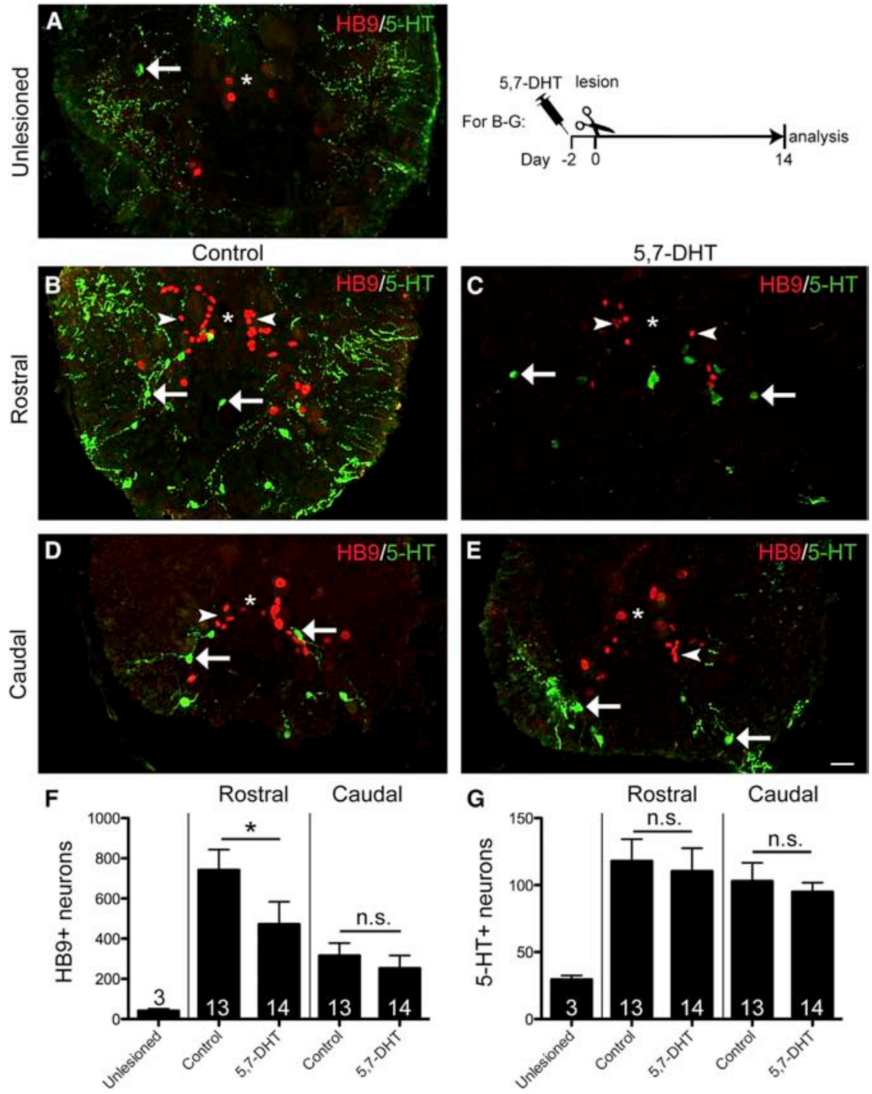

Figure 4. Ablation of Serotonergic Axons Inhibits Regeneration of Motor Neurons, but Not Serotonergic Neurons

Spinal cross-sections are shown (dorsal is up; asterisk indicates central canal).

(A) In the unlesioned spinal cord, few small HB9$^+$ and motor neurons and 5-HT$^+$ cells (arrow) are present.

(B–G) Rostral to the lesion (see timeline for experimental condition), ablation of serotonergic axons leads to reduced motor neuron (arrowheads) regeneration without influencing regeneration of serotonergic neurons (arrows). Caudal to the lesion, numbers of newly generated motor neurons and serotonergic neurons are unaltered (Mann-Whitney U-test; *p = 0.0344). The scale bar in (E) represents 25 μm.

See also Figure S4.

regeneration of motor neurons can be substituted by exogenous serotonin in the spinal cord caudal to a lesion, a region that is deprived of descending serotonergic axons.

To determine whether increasing the serotonin signal in the absence of a spinal lesion was sufficient to induce motor neuron or serotonergic interneuron generation, we injected unlesioned animals with serotonin in the same way as lesioned animals. However, the very low number of small HB9$^+$ neurons (control: n = 8, 60.25 ± 10.97; serotonin treated: n = 6, 59.27 ± 10.44; p = 1) as well as that of serotonergic neurons (control: n = 8, 30.99 ± 4.35; serotonin treated: n = 6, 28.84 ± 4.94; p = 0.7546) remained unchanged. This indicates that a lesion signal is needed to make pMN cells competent to react to the serotonin signal.

Serotonin Increases Lesion-Induced Proliferation in Adult pMNs

Caspase 3 labeling indicated that the effect of serotonin was unlikely on motor neuron survival (see above and Figures S4F–S4I). To determine whether serotonin acted on pMN-like ERGs, we injected lesioned olig2:GFP fish with serotonin at 3, 6, and 9 dpl and analyzed ventricular proliferation at 10 dpl using PCNA and EdU labeling (Figures 5F and 5G). Compared to controls (vehicle-injected lesioned animals), both markers were increased in pMN-like ERGs (PCNA: 94%; EdU: 71%; Figures 5H and 5I). Activation of proliferation in the pMN-like ERG domain caudal to the lesion only is consistent with the rostro-caudal differences in motor neuron generation. Interestingly, outside the pMN-like domain, proliferation was not significantly changed (Figures S5G and S5H). In unlesioned animals, serotonin injections had no effect on proliferative events in the pMN-like domain (PCNA; control: n = 5, 4.49 ± 2.79 cells/750 μm; serotonin treated: n = 4, 9.55 ± 4.13; p = 0.4428). These observations support that pMN-like ERGs are selectively sensitive to the serotonin signal but only become competent to react to it after a lesion.

DISCUSSION

We demonstrate that serotonin promotes embryonic development and adult regeneration of motor neurons. Serotonin increases proliferation in both embryonic pMN progenitors and adult pMN-like ERGs. Progenitor types in the spinal cord react differently to the serotonin signal, as generation of interneuron populations during development and lesion-induced generation of serotonergic interneurons in adults were not altered by experimental manipulations of serotonin.

Another monoamine signal that stimulates motor neuron development and regeneration is dopamine (Reimer et al., 2013), which is derived from diencephalic neurons in both development and regeneration (Kuscha et al., 2012a; McLean and

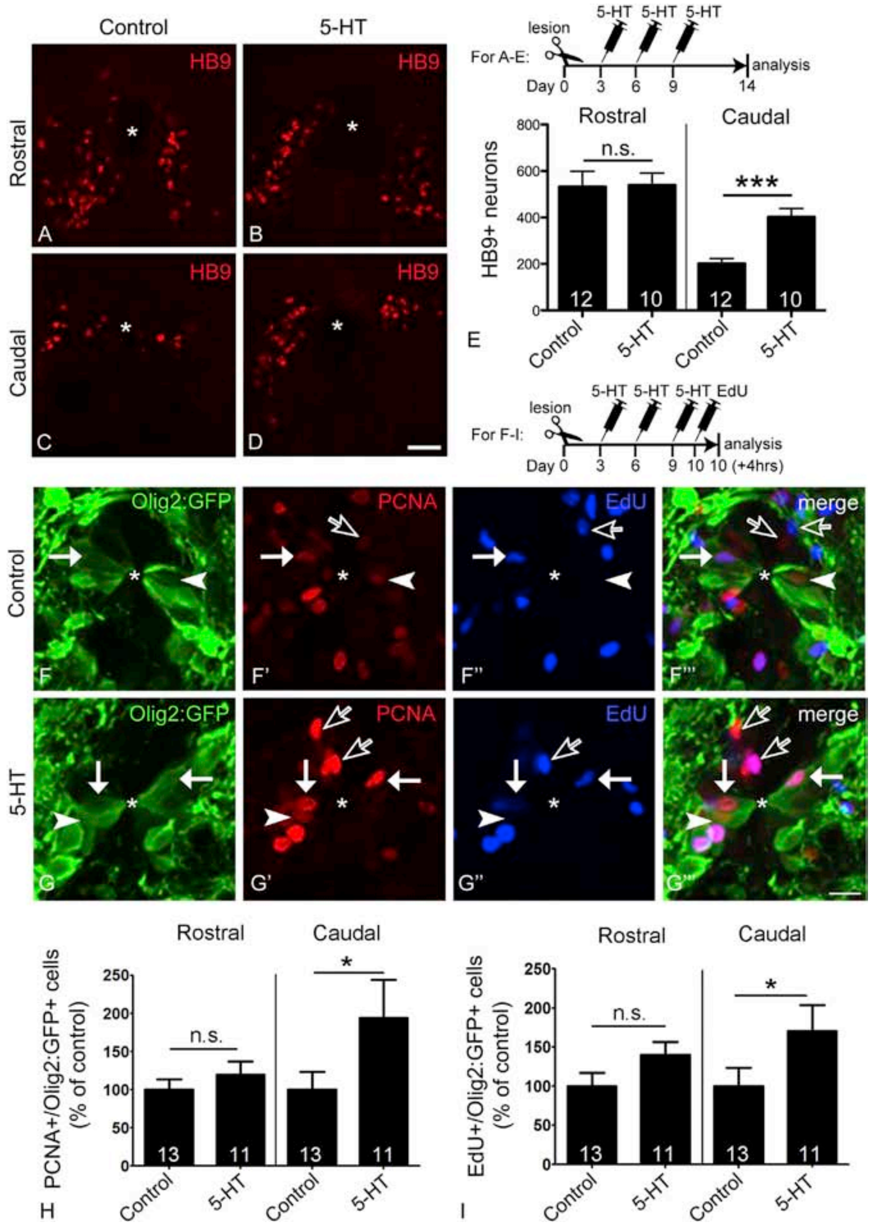

Figure 5. Serotonin Injections Increase the Number of Newly Generated Motor Neurons and Proliferating pMN-like Progenitor Cells Caudal to the Spinal Lesion

Cross-sections through the spinal cord are shown; asterisks indicate the central canal, arrowheads indicate PCNA$^+$/olig2:GFP$^+$ cells, solid arrows indicate EdU$^+$/olig2:GFP$^+$ cells, and empty arrows indicate PCNA$^+$/olig2:GFP$^-$ or EdU$^+$/olig2:GFP$^-$ cells.

(A–E) Serotonin injection doubles the number of newly generated motor neurons caudal to the lesion but has no effect rostral to the lesion (see timeline in E for experimental paradigm; Student's t test; ***p < 0.0001).

(F–I) In the ventricular zone of olig2:GFP transgenic animals, the numbers of PCNA$^+$/olig2:GFP$^+$ (Mann-Whitney U-test; *p = 0.0437) and EdU$^+$/olig2:GFP$^+$ (Mann-Whitney U-test; *p = 0.0231) pMN-like ERGs are significantly increased only caudal to the lesion (see timeline for experimental paradigm).

The scale bar in (D) represents 50 μm for (A)–(D) and in (G''') represents 10 μm for (F) and (G). See also Figure S5.

Fetcho, 2004). In contrast, serotonin appears to be diffusely distributed in embryos, whereas in the adult spinal cord, it is mainly derived from descending brainstem axons (Kuscha et al., 2012a; Lillesaar et al., 2009; McLean and Fetcho, 2004). Another interesting difference is that dopamine attenuates developmental generation of V2 interneurons, whereas serotonin does not influence generation of these interneurons.

Both monoamines likely promote generation of motor neurons independently of each other. In embryos with pharmacologically blocked dopamine signaling, serotonin still promotes motor neuron generation. In lesioned adults, dopamine or serotonin alone promote motor neuron generation caudal to the spinal lesion in the absence of either intact descending dopaminergic or serotonergic axons, as both descending axon types completely degenerate caudal to the lesion (Kuscha et al., 2012a). In addition, ablation of serotonergic axons did not alter spinal dopamine immuno-labeling in the adult spinal cord rostral to the lesion, further supporting that serotonin does not act indirectly through increasing dopamine signaling.

Serotonin likely acts by promoting progenitor proliferation in development and adult regeneration, as addition of serotonin increased the number of proliferating pMN progenitors in embryos. In adults, a doubling of proliferating pMN-like ERGs occurred only caudal to the lesion, which corresponded to a doubling of motor neuron numbers only there. The observation that outside the pMN-like domain no significant increase in proliferation of ventricular cells was elicited suggests that the serotonin signal is relatively selective for pMN-like ERGs. However, we cannot exclude that serotonin acts on neuroblast differentiation in addition to progenitor cell proliferation.

In accordance with the observed increase in progenitor proliferation, pMN progenitors in embryos showed expression of a large number of serotonin receptors. Evidence from treatment with 5-HT1A-specific agonists and antagonists and knockdown of *htr1ab* expression suggests activity of at least this receptor in motor neuron development. Interestingly, adult pMN-like ERGs express *htr1aa*, but not *htr1ab*. This could indicate a switch in receptor usage in adult regeneration. Regeneration-specific gene expression has been observed in zebrafish (Dias et al., 2012; Kizil et al., 2012b). Interestingly, the mammalian homolog *Htr1a* promotes adult neurogenesis in the brain (Banasr et al., 2004).

Ependymal cells in spinal-lesioned lampreys also express *htr1a* (Cornide-Petronio et al., 2014). However, several serotonin receptors could be involved in motor neuron regeneration. Of note, adding serotonin in the absence of a lesion is insufficient to induce proliferation or motor neuron generation, indicating that progenitors have to be in a lesion-induced sensitized state to become competent to react to serotonin.

Our present and recent results (Reimer et al., 2013) indicate that the rostral part of the lesioned spinal cord presents a significantly different environment from that in the caudal spinal cord. Regeneration-promoting descending dopaminergic and serotonergic axons present rostral, but not caudal, to a lesion, where these axons degenerate. Local serotonergic neurons in the caudal spinal cord cannot adequately compensate for the lack of descending axons, even though they increase in number after a spinal lesion. That is because their terminals account for only 7% of serotonergic terminals present rostral to the lesion and are rarely close to ERG processes (Figures 2E and 2F). Accordingly, we find twice as many newly generated motor neurons rostral than caudal to the lesion. This difference is not due to intrinsic rostro-caudal differences in the spinal cord, as this phenomenon is independent of the position of the lesion. How descending axons influence post-lesion events in other species needs to be determined.

However, serotonergic innervation from the brain does not influence the lesion-induced generation of serotonergic interneurons, similar to the lack of effect on interneuron types during development. Serotonergic cells are generated in equal numbers rostral and caudal to the lesion despite a much stronger serotonergic innervation rostral to the lesion, and experimental manipulations of the serotonin signal do not influence the number of serotonergic neurons. Thus, progenitors for serotonergic neurons, located ventral to pMNs (Kuscha et al., 2012a), are insensitive to serotonin itself. In contrast, in the hypothalamus of adult zebrafish, serotonin provides positive feedback for the generation of serotonergic neurons (Pérez et al., 2013). Conversely, in the salamander midbrain, dopamine has been found to inhibit regeneration of dopaminergic neurons, thus providing a mechanism by which regeneration is self-limiting (Berg et al., 2011). Our results indicate that serotonin levels in the spinal cord do not control numbers of regenerating serotonergic neurons there. Rather, serotonergic neurons are strongly over-produced after a lesion and only later pruned back in number (Kuscha et al., 2012a). The same is true for motor neurons—only a quarter of the newly generated motor neurons mature, with the rest being eliminated (Reimer et al., 2008). Thus, control of lesion-induced and homeostatic neurogenesis differs between regenerating species, CNS regions, and progenitor type.

In mammals, ependymal cells, which are related to ERGs in fish, proliferate and contribute cells to the glial scar after spinal cord injury, but no new neurons are generated (Meletis et al., 2008). Viral overexpression of transcription factors drives newly generated spinal cells toward neuronal identity (Ohori et al., 2006; Su et al., 2014), but motor neurons are not induced. We show here that, in an adult vertebrate, serotonin significantly increases the number of newly generated motor neurons after a spinal lesion and that exogenous serotonin can replace the endogenous signal.

EXPERIMENTAL PROCEDURES

Animals

All fish were kept and bred in our laboratory fish facility according to standard methods (Westerfield, 2000), and all experiments have been approved by the British Home Office. For a list of lines used, see Supplemental Experimental Procedures.

Drug and Morpholino Treatments

In embryos, drugs were added to the medium from 24 hpf and morpholinos were injected into fertilized eggs, as described (Reimer et al., 2013). Adult fish were anesthetized and injected with substances intraperitoneally at a volume of 25 μl. See Supplemental Experimental Procedures for detail.

Immunohistochemistry and Quantifications

A list of antibodies and reagents used can be found in the Supplemental Experimental Procedures. Immunohistochemistry on embryo whole mounts and section of the adult spinal cord have been described (Reimer et al., 2013). In embryos, motor neurons and other cell types were counted in confocal image stacks of midthoracic segments, or the percentage of embryos in which spinal islet-1:GFP^{+} motor neurons were present were scored, as described (Reimer et al., 2013). In sections of adult spinal cord, stereological cell counts and fiber counts were performed as described (Kuscha et al., 2012a). All quantifications were done without knowledge of the experimental condition of the specimens. See Supplemental Experimental Procedures for full detail and statistical methods.

Fluorescence-Activated Cell Sorting and RT-PCR

Motor neurons and pMN cells were isolated from double-transgenic olig2:DsRed/HB9:GFP embryos by FACS of cells from dissociated embryos followed by RT-PCR as described (Reimer et al., 2013). To isolate pMN-like progenitors from the adult spinal cord, we isolated olig2:dsRed^{+}/mbp:GFP^{-} cells from double-transgenic animals. See Supplemental Experimental Procedures for detailed protocols and primers used.

Spinal Cord Lesion

As described previously (Becker et al., 1997), fish were anesthetized by immersion in 0.02% aminobenzoic acid ethyl methyl ester (MS222; Sigma) in PBS for 5 min. A longitudinal incision was made at the side of the fish to expose the vertebral column. The spinal cord was completely transected under visual control, 3.5 mm caudal to the brainstem-spinal cord junction if not indicated differently.

SUPPLEMENTAL INFORMATION

Supplemental Information includes Supplemental Experimental Procedures and five figures and can be found with this article online at http://dx.doi.org/10.1016/j.celrep.2015.09.050.

AUTHOR CONTRIBUTIONS

A.B.-I., K.S.M., and A.L.S. performed most of the experiments, analyzed data, and critically contributed to manuscript preparation. M.M.R. and Y.Y. contributed experimental data and discussions. C.G.B. and T.B. contributed experiments, oversaw the study, and wrote the manuscript.

ACKNOWLEDGMENTS

We thank Dr. Laure Bally-Cuif for probes and transgenic tools; David Lyons, Hitoshi Okamoto, Bruce Appel, Dirk Meyer, Shin-ishi Higashijima, and Michael Brand for transgenic fish; and Dr. Claire Cryer and Mrs. Fiona Rossi for assistance with FACS. Supported by the BBSRC (BB/I01294X/1), postdoctoral fellowships from the Fundación Barrié (Spain) and Xunta de Galicia (Spain) to A.B.-I., an EMBO fellowship to A.L.S., and a BBSRC DTG PhD studentship to K.S.M.

Received: April 3, 2014
Revised: June 13, 2015
Accepted: September 17, 2015
Published: October 22, 2015

REFERENCES

Almeida, R.G., Czopka, T., Ffrench-Constant, C., and Lyons, D.A. (2011). Individual axons regulate the myelinating potential of single oligodendrocytes in vivo. Development *138*, 4443–4450.

Banasr, M., Hery, M., Printemps, R., and Daszuta, A. (2004). Serotonin-induced increases in adult cell proliferation and neurogenesis are mediated through different and common 5-HT receptor subtypes in the dentate gyrus and the subventricular zone. Neuropsychopharmacology *29*, 450–460.

Bashammakh, S., Würtele, M., Kotnik, K., Abdelilah-Seyfried, S., and Bader, S. (2014). Serotonin is required for pharyngeal arch morphogenesis in zebrafish (ScienceOpen Research), Published online December 22, 2014. http://dx.doi.org/10.14293/S2199-1006.1.SOR-LIFE.AWPDLZ.v1.

Becker, C.G., and Becker, T. (2015). Neuronal regeneration from ependymo-radial glial cells: cook, little pot, cook!. Dev. Cell *32*, 516–527. http://dx.doi.org/10.1016/j.devcel.2015.01.001.

Becker, T., Wullimann, M.F., Becker, C.G., Bernhardt, R.R., and Schachner, M. (1997). Axonal regrowth after spinal cord transection in adult zebrafish. J. Comp. Neurol. *377*, 577–595.

Berg, D.A., Kirkham, M., Wang, H., Frisén, J., and Simon, A. (2011). Dopamine controls neurogenesis in the adult salamander midbrain in homeostasis and during regeneration of dopamine neurons. Cell Stem Cell *8*, 426–433.

Berg, D.A., Belnoue, L., Song, H., and Simon, A. (2013). Neurotransmitter-mediated control of neurogenesis in the adult vertebrate brain. Development *140*, 2548–2561.

Choi, S., Jonak, E., and Fernstrom, J.D. (2004). Serotonin reuptake inhibitors do not prevent 5,7-dihydroxytryptamine-induced depletion of serotonin in rat brain. Brain Res. *1007*, 19–28.

Cole, L.K., and Ross, L.S. (2001). Apoptosis in the developing zebrafish embryo. Dev. Biol. *240*, 123–142.

Cornide-Petronio, M.E., Fernández-López, B., Barreiro-Iglesias, A., and Rodicio, M.C. (2014). Traumatic injury induces changes in the expression of the serotonin 1A receptor in the spinal cord of lampreys. Neuropharmacology *77*, 369–378.

Dessaud, E., McMahon, A.P., and Briscoe, J. (2008). Pattern formation in the vertebrate neural tube: a sonic hedgehog morphogen-regulated transcriptional network. Development *135*, 2489–2503.

Dias, T.B., Yang, Y.J., Ogai, K., Becker, T., and Becker, C.G. (2012). Notch signaling controls generation of motor neurons in the lesioned spinal cord of adult zebrafish. J. Neurosci. *32*, 3245–3252.

Doze, V.A., and Perez, D.M. (2012). G-protein-coupled receptors in adult neurogenesis. Pharmacol. Rev. *64*, 645–675.

Fan, L.W., Bhatt, A., Tien, L.T., Zheng, B., Simpson, K.L., Lin, R.C., Cai, Z., Kumar, P., and Pang, Y. (2015). Exposure to serotonin adversely affects oligodendrocyte development and myelination in vitro. J. Neurochem. *133*, 532–543.

Goldshmit, Y., Sztal, T.E., Jusuf, P.R., Hall, T.E., Nguyen-Chi, M., and Currie, P.D. (2012). Fgf-dependent glial cell bridges facilitate spinal cord regeneration in zebrafish. J. Neurosci. *32*, 7477–7492.

Jiménez-Trejo, F., Tapia-Rodríguez, M., Cerbón, M., Kuhn, D.M., Manjarrez-Gutiérrez, G., Mendoza-Rodríguez, C.A., and Picazo, O. (2012). Evidence of 5-HT components in human sperm: implications for protein tyrosine phosphorylation and the physiology of motility. Reproduction *144*, 677–685.

Kizil, C., Kaslin, J., Kroehne, V., and Brand, M. (2012a). Adult neurogenesis and brain regeneration in zebrafish. Dev. Neurobiol. *72*, 429–461.

Kizil, C., Kyritsis, N., Dudczig, S., Kroehne, V., Freudenreich, D., Kaslin, J., and Brand, M. (2012b). Regenerative neurogenesis from neural progenitor cells requires injury-induced expression of Gata3. Dev. Cell *23*, 1230–1237.

Kuscha, V., Barreiro-Iglesias, A., Becker, C.G., and Becker, T. (2012a). Plasticity of tyrosine hydroxylase and serotonergic systems in the regenerating spinal cord of adult zebrafish. J. Comp. Neurol. *520*, 933–951.

Kuscha, V., Frazer, S.L., Dias, T.B., Hibi, M., Becker, T., and Becker, C.G. (2012b). Lesion-induced generation of interneuron cell types in specific dorso-ventral domains in the spinal cord of adult zebrafish. J. Comp. Neurol. *520*, 3604–3616.

Lauder, J.M., and Krebs, H. (1978). Serotonin as a differentiation signal in early neurogenesis. Dev. Neurosci. *1*, 15–30.

Lillesaar, C., Stigloher, C., Tannhäuser, B., Wullimann, M.F., and Bally-Cuif, L. (2009). Axonal projections originating from raphe serotonergic neurons in the developing and adult zebrafish, Danio rerio, using transgenics to visualize raphe-specific pet1 expression. J. Comp. Neurol. *512*, 158–182.

McLean, D.L., and Fetcho, J.R. (2004). Ontogeny and innervation patterns of dopaminergic, noradrenergic, and serotonergic neurons in larval zebrafish. J. Comp. Neurol. *480*, 38–56.

Meletis, K., Barnabé-Heider, F., Carlén, M., Evergren, E., Tomilin, N., Shupliakov, O., and Frisén, J. (2008). Spinal cord injury reveals multilineage differentiation of ependymal cells. PLoS Biol. *6*, e182.

Myers, P.Z., Eisen, J.S., and Westerfield, M. (1986). Development and axonal outgrowth of identified motoneurons in the zebrafish. J. Neurosci. *6*, 2278–2289.

Ohori, Y., Yamamoto, S., Nagao, M., Sugimori, M., Yamamoto, N., Nakamura, K., and Nakafuku, M. (2006). Growth factor treatment and genetic manipulation stimulate neurogenesis and oligodendrogenesis by endogenous neural progenitors in the injured adult spinal cord. J. Neurosci. *26*, 11948–11960.

Pérez, M.R., Pellegrini, E., Cano-Nicolau, J., Gueguen, M.M., Menouer-Le Guillou, D., Merot, Y., Vaillant, C., Somoza, G.M., and Kah, O. (2013). Relationships between radial glial progenitors and 5-HT neurons in the paraventricular organ of adult zebrafish - potential effects of serotonin on adult neurogenesis. Eur. J. Neurosci. *38*, 3292–3301.

Reimer, M.M., Sörensen, I., Kuscha, V., Frank, R.E., Liu, C., Becker, C.G., and Becker, T. (2008). Motor neuron regeneration in adult zebrafish. J. Neurosci. *28*, 8510–8516.

Reimer, M.M., Kuscha, V., Wyatt, C., Sörensen, I., Frank, R.E., Knüwer, M., Becker, T., and Becker, C.G. (2009). Sonic hedgehog is a polarized signal for motor neuron regeneration in adult zebrafish. J. Neurosci. *29*, 15073–15082.

Reimer, M.M., Norris, A., Ohnmacht, J., Patani, R., Zhong, Z., Dias, T.B., Kuscha, V., Scott, A.L., Chen, Y.C., Rozov, S., et al. (2013). Dopamine from the brain promotes spinal motor neuron generation during development and adult regeneration. Dev. Cell *25*, 478–491.

Su, Z., Niu, W., Liu, M.L., Zou, Y., and Zhang, C.L. (2014). In vivo conversion of astrocytes to neurons in the injured adult spinal cord. Nat. Commun. *5*, 3338.

Valencia, C.A., Bailey, C., and Liu, R. (2007). Novel zebrafish caspase-3 substrates. Biochem. Biophys. Res. Commun. *361*, 311–316.

Westerfield, M. (2000). The Zebrafish Book: A Guide for the Laboratory Use of Zebrafish (*Danio rerio*), Fourth Edition (Eugene: University of Oregon Press).

Zeng, C., Pan, F., Jones, L.A., Lim, M.M., Griffin, E.A., Sheline, Y.I., Mintun, M.A., Holtzman, D.M., and Mach, R.H. (2010). Evaluation of 5-ethynyl-2′-deoxyuridine staining as a sensitive and reliable method for studying cell proliferation in the adult nervous system. Brain Res. *1319*, 21–32.

TITLE: Serotonin promotes development and regeneration of spinal motor neurons in zebrafish.

AUTHORS: Antón Barreiro-Iglesias, Karolina S. Mysiak, Angela L. Scott, Michell M. Reimer, Yujie Yang (杨宇婕), Catherina G. Becker, Thomas Becker

SUPPLEMENTAL EXPERIMENTAL PROCEDURES

Animals

We used wild type (*wik*); *Tg(mnx1:GFP)*^{ml2} abbreviated as HB9:GFP (Flanagan-Steet et al., 2005); *Tg(olig2:EGFP),* abbreviated as olig2:GFP (Shin et al., 2003); *Tg(olig2:DsRed2),* abbreviated as olig2:dsRed (Kucenas et al., 2008); *Tg(pax2a:GFP)* abbreviated as pax2a:GFP (Picker et al., 2002); *TgBAC(vsx1:GFP)* abbreviated as vsx1:GFP (Kimura et al., 2008); and *Tg(mbp:EGFP)*, abbreviated as mbp:GFP (Almeida et al., 2011) transgenic lines. Male and female fish were used for the experiments.

Antibodies and reagents

We used mouse anti-HB9 (MNR2, 1:400, Developmental Studies Hybridoma Bank), rabbit anti-serotonin (1:2500, Sigma), mouse anti-PCNA (1:1000, Dako), rabbit anti-pH3 (1:1000, Millipore), chicken anti-GFP (1:500, Abcam), mouse anti-TH1 (1:1000, Millipore) rabbit anti-dopamine (1:750, Dr. H.W.M. Steinbusch, Maastricht University, The Netherlands) and rabbit anti-active caspase 3 (1:500, BD Pharmigen, Cat. No. 559565). Secondary antibodies were donkey anti-mouse Cy3, goat anti-rabbit Dylight-488, donkey anti-chicken Alexa-488 and donkey anti-rabbit Cy3 (all 1:200, Jackson

ImmunoResearch). EdU was detected according to the manufacturer's protocol using Alexa-647 azide as Component B.

Immunohistochemistry and cell quantification in embryos

Immunohistochemistry on embryo whole-mounts has been described (Reimer et al., 2013). Motor neurons and other cell types were counted in confocal image stacks of two or four (pH3 counts only) midthoracic segments as described (Reimer et al., 2013). In some experiments, the percentage of embryos in which spinal islet-1:GFP+ motor neurons were present were scored relative to controls. This was done at least in triplicate with at least 15 embryos per treatment group. This test measures promotion or inhibition of islet-1:GFP+ motor neuron development in a dose-dependent fashion, as confirmed by cell counts and fluorescence intensity measurement (Reimer et al., 2013). For all quantifications, the observer was blinded to the experimental treatments.

Immunohistochemistry and quantifications in adults

Immunohistochemistry on 50 µm vibrating blade microtome sections has been described (Kuscha et al., 2012). Quantification of serotonin, TH1 or dopamine immuno-reactive varicosities in confocal images was done semi-automatically as previously described (Kuscha et al., 2012), except that particles were counted with a particle size from 0 to infinity in Image J (comparing this method with manual counts showed a very good correspondence). Quantification of dopamine immunofluorescence intensity was done with Image J and is given as the mean intensity and the number of

pixels of the highest intensity (255) per spinal cord section (3 sections were analyzed per fish). The observer was blinded to the experimental groups during quantifications.

Cell numbers rostral and caudal to the lesion site were determined in a blinded fashion from stereological counts of confocal images as previously described (Reimer et al., 2013) and schematically depicted in Fig. S1E.

Drug and morpholino treatments in embryos

Drugs (serotonin hydrochloride (5-HT; Sigma H9523), 4-Chloro-DL-phenylalanine (pCPA; Sigma C6506), (±)-8-Hydroxy-2-(dipropylamino)tetralin hydrobromide (8-OH-DPAT; Sigma H8520), fluvoxamine maleate (Sigma F2802), WAY100635 maleate salt (Sigma W108), L-745,870 trihydrochloride (Tocris Bioscience 1002) and pergolide mesylate salt (Sigma P8828) were added to the embryos from 24 hpf at a standard concentration of 10 μM (50 μM for pCPA), as described (Reimer et al., 2013). The *htr1ab* morpholino (5'-ACGTGTCGTTGTTTTCTTCCATGTC -3') and control morpholino (5'-CCTCTTACCTCAGTTACAATTTATA -3') were injected at a concentration of 0.5 mM into fertilized eggs, as described (Reimer et al., 2013).

Intraperitoneal substance applications in adults

Animals were anesthetized and injected intraperitoneally at a volume of 25 μl. 5,7-dihydroxytryptamine (5,7-DHT, Sigma) was dissolved in distilled water and injected at a concentration of 4 mg/ml. Serotonin hydrochloride was dissolved in distilled water and injected at a concentration of 0.2 mg/ml. 5-

ethynyl-2'-deoxyuridine (EdU; InVitrogen, Paisley, UK) was dissolved in 0.3 x Danieau's solution with 15% DMSO to 4.11 mg/ml. Vehicle injections served as controls.

Fluorescence activated cell sorting and RT-PCR in embryos

Motor neurons and pMN cells (25000 and 17000 cells respectively from 200 embryos) were isolated from double transgenic olig2:DsRed/HB9:GFP animals by lysing the embryos and FAC sorting of cells followed by determination of relative receptor expression levels by RT-PCR as described (Reimer et al., 2013). We used the following primers to amplify serotonin receptor sequences: *htr1aa* forward: 5'-GCTTCGAGAACCGAAATGAG-3'; *htr1aa* reverse: 5'-GCTACGATGAAGAAGGGCAG-3'; *htr1ab* forward: 5'-GAGCAAAAACTGGAAAAGCG-3'; *htr1ab* reverse: 5'-GCAGCCAACACAGAATGAAA-3'; *htr1b* forward: 5'-ATCATCGCCATCTCCATCTC-3'; *htr1b* reverse: 5'-GCAGAGGTGAGTCTTTTGCC-3'; *htr1bd* forward: 5'-TGTTCAACAGCTCCGATGAG-3'; *htr1bd* reverse: 5'-AATGGACACCAGGAGGTCTG-3'; *htr1e* forward: 5'-CATATACAGCGCTGCCAAGA-3'; *htr1e* reverse: 5'-GACACAGAAGGCATGCTTGA-3'; *htr2ab* forward: 5'-TTCTGGTCAACACAATCCCA-3'; *htr2ab* reverse: 5'-GCTTTTGGACAGATGGTGGT-3'; *htr3a* forward: 5'-CCAGAGCTGGCTACACACAA-3'; *htr3a* reverse: 5'-GTGAAGCAGCTCCCATTCTC-3'; *htr3b* forward: 5'-AATATCGCTGTCCTCGGATG-3'; *htr3b* reverse: 5'-

TCCACTTCGCTCACTGAATG-3'; *htr4* forward: 5'-CATGGTGAACAAGCCATACG-3'; *htr4* reverse: 5'-CCACCACGTTTGTGATGAAG-3'; *htr5a* forward: 5'-CGACACCTGGAGTACACCCT-3'; *htr5a* reverse: 5'-TCCTCCGAATACGTCTCACC-3'; *htr7a* forward: 5'-GAGAAAGTGCTCATCGGAGG-3'; *htr7a* reverse: 5'-CGGTACAGCACATCACATCC-3'.

For amplification of control sequences we used the following primers: *olig2* forward: 5'-AATGAGCTGCAGAGCATGCG-3'; *olig2* reverse: 5'-TAAGAGTGCCACAACTGGACGG-3'; *dsRed* forward: 5'-AGTTCCAGTACGGCTCCAAG-3'; *dsRed* reverse: 5'-TTGTGGGTCTCGCCCTTCA-3'; *GAPDH* forward: 5'-ACTCCACTCATGGCCGTTAC-3'; *GAPDH* reverse: 5'-TCTTCTGTGTGGCGGTGTAG-3'.

FAC sorting and PCR of adult pMN-like ERGs

For the purification of olig2:dsRed$^+$/MBP:GFP$^-$ cells, we dissociated unlesioned spinal cords from 4 adult olig2:dsRed x MBP:GFP fish, yielding 2041 dsRed$^+$/GFP$^-$ and 941 dsRed$^+$/GFP$^+$ cells. In addition, 2 spinal cords from each WIK, olig2:dsRed, and MBP:GFP lines were used for the calibration of the FAC sorter. The fish were perfused with cold PBS. Spinal cords were dissected and washed twice in calcium-free Ringer buffer. Then the solution was removed and the tissue was minced with a scalpel. The tissue was incubated in EDTA-trypsin (0.25% trypsin and 1mM EDTA in PBS) at room temperature with very gentle agitation for 45 min, followed by

trituration with a pipette. The dissociation was stopped by addition of equal volume of 2 mM CaCl2 with 20% fetal calf serum (FCS) and centrifugation at 3500 g for 5 min. The cells were resuspended in 1ml 2% FCS/2mM EDTA in PBS. The samples were sorted using the BD FACSAria II Flow Cytometer (BD Biosciences, San Jose, California, USA). Live cells were distinguished from debris using DRAQ5 and DAPI staining. The purity of the isolated cells was between 86-97%. Cells were immediately processed for RNA extraction and first-strand cDNA synthesis using Cells Direct One-Step qRT-PCR Kit (Invitrogen). To amplify *EF1* α we used forward: 5'-AGGACATCCGTCGTGGTAAT-3'; reverse: 5'-AGAGATCTGACCAGGGTGGTT-3' primers.

Statistical analyses

Variability of values is always given as SEM. Statistical significance was determined using Student's t-test (for normally distributed data) or Mann-Whitney U-test. We used ANOVA with appropriate post-tests for multiple comparisons.

Literature cited for supplemental experimental procedures

Almeida, R.G., Czopka, T., Ffrench-Constant, C., and Lyons, D.A. (2011). Individual axons regulate the myelinating potential of single oligodendrocytes in vivo. Development *138*, 4443-4450.

Flanagan-Steet, H., Fox, M.A., Meyer, D., and Sanes, J.R. (2005). Neuromuscular synapses can form in vivo by incorporation of initially aneural postsynaptic specializations. Development *132*, 4471-4481.

Kimura, Y., Satou, C., and Higashijima, S. (2008). V2a and V2b neurons are generated by the final divisions of pair-producing progenitors in the zebrafish spinal cord. Development *135*, 3001-3005.

Kucenas, S., Takada, N., Park, H.C., Woodruff, E., Broadie, K., and Appel, B. (2008). CNS-derived glia ensheath peripheral nerves and mediate motor root development. Nat Neurosci *11*, 143-151.

Kuscha, V., Barreiro-Iglesias, A., Becker, C.G., and Becker, T. (2012). Plasticity of tyrosine hydroxylase and serotonergic systems in the regenerating spinal cord of adult zebrafish. J Comp Neurol *520*, 933-951.

Picker, A., Scholpp, S., Bohli, H., Takeda, H., and Brand, M. (2002). A novel positive transcriptional feedback loop in midbrain-hindbrain boundary development is revealed through analysis of the zebrafish pax2.1 promoter in transgenic lines. Development *129*, 3227-3239.

Reimer, M.M., Norris, A., Ohnmacht, J., Patani, R., Zhong, Z., Dias, T.B., Kuscha, V., Scott, A.L., Chen, Y.C., Rozov, S.*, et al.* (2013). Dopamine from the Brain Promotes Spinal Motor Neuron Generation during Development and Adult Regeneration. Dev Cell *25*, 478-491.

Shin, J., Park, H.C., Topczewska, J.M., Mawdsley, D.J., and Appel, B. (2003). Neural cell fate analysis in zebrafish using olig2 BAC transgenics. Methods Cell Sci *25*, 7-14.

SUPPLEMENTAL FIGURES

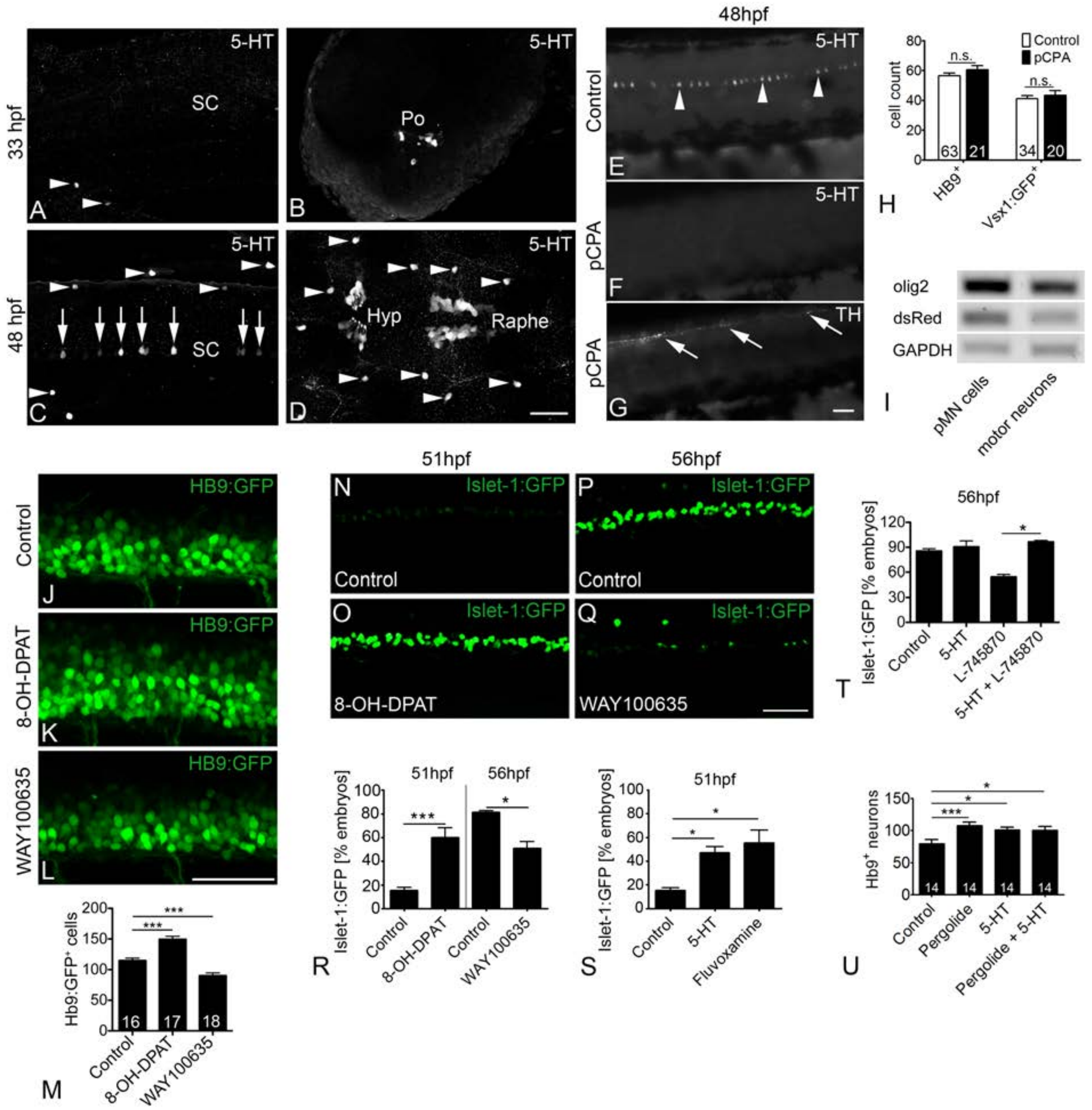

Fig. S1 (related to Fig. 1). Serotonin (5-HT) promotes motor neuron development. **A,C:** Lateral views of the trunk region (left column) indicate that spinal-intrinsic serotonergic cells (arrows) are present at 48, but not 33 hpf. (arrowheads indicate serotonergic cells in the periphery). **B:** A dorsal view of the brain indicates only a few serotonergic cells in the region of the preoptic nucleus (Po) of the diencephalon. **D:** In contrast, large populations of

serotonin-labeled neurons are present in dorsal views in the hypothalamus (Hyp) and raphe region at 48 hpf. **E-G:** Lateral views of the trunk indicate that treatment with pCPA from 24 hpf effectively inhibits serotonin (5-HT) accumulation in spinal neurons (E,F), but does not influence the presence of tyrosine hydroxylase (TH) expressing descending axons (arrows) in the spinal cord (G). **H:** pCPA treatment does not affect numbers of motor neurons or vsx1:GFP+ interneurons at 33 hpf. **I:** Endogenous *olig2* mRNA, as well as *DsRed* mRNA are enriched in FAC sorted progenitor cells, compared to the motor neuron fraction, as shown by RT-PCR in olig2:DsRed and HB9:GFP double transgenic fish. GAPDH is used for comparison. **J-M:** A 5-HT1A agonist (8OH-DPAT) increases and an antagonist (WAY100645) decreases numbers of hb9:GFP+ motor neurons (ANOVA, $P<0.0001$). Lateral views of embryos at trunk level are shown; drug incubation form 24 to 33 hpf. **N-R:** Similarly, 8-OH-DPAT promotes (Student t-test, $P=0.0002$) and WAY100645 inhibits (Mann-Whitney U-test, one-tailed, $P=0.0383$) development of islet-1:GFP+ motor neurons. **S:** Serotonin re-uptake inhibitor fluvoxamine promotes development of islet-1:GFP+ motor neurons, similar to serotonin (ANOVA with Dunn's Multiple Comparison Test, $p < 0.05$). **T:** Serotonin rescues the effect of blocking Drd4 receptors with L-745870 on development of islet-1:GFP+ motor neurons (Mann-Whitney U-test, one-tailed, $p=0.0383$), indicating that serotonin does not act through the Drd4a receptor. **U:** Dopamine agonist pergolide (ANOVA with Bonferroni's Multiple Comparison Test, $p < 0.001$) and serotonin ($p < 0.05$) increase numbers of HB9+ motor neurons, but have no additive effect. Scale bars = 50 μm.

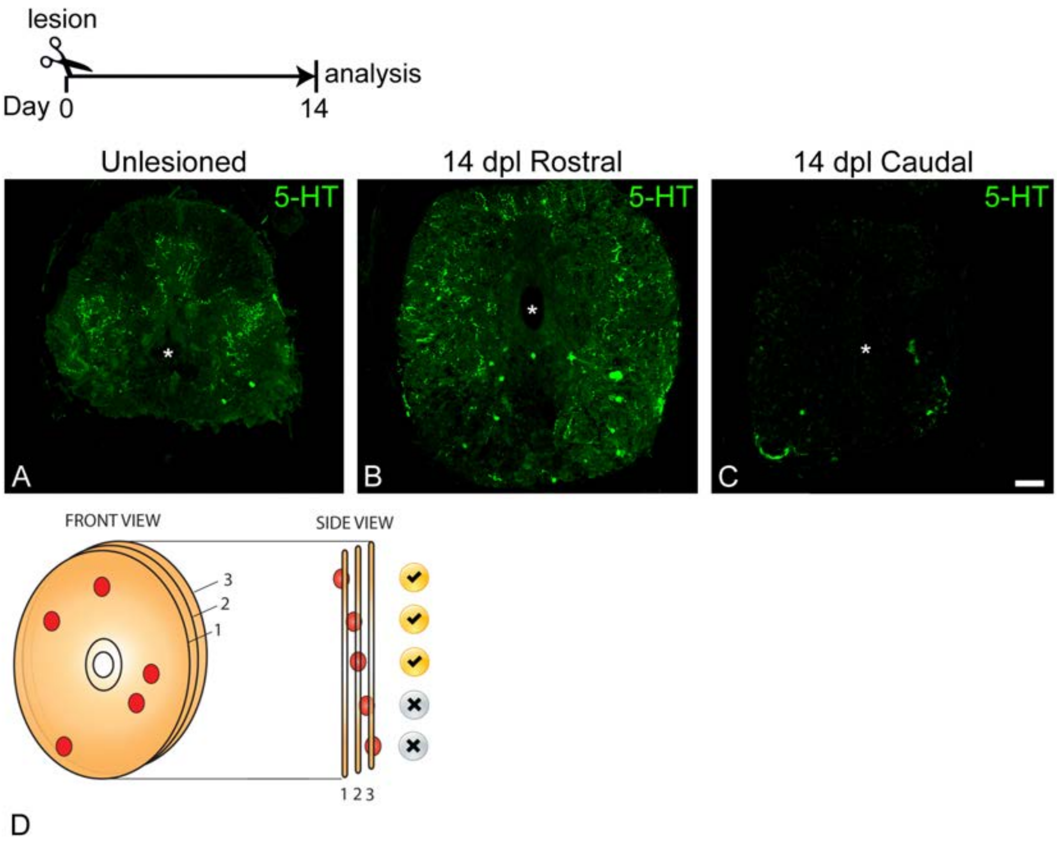

Fig. S2 (related to Fig. 2) Characterization of serotonergic (5-HT) signal and pMN progenitors. **A-C:** Distribution of serotonergic axons in the unlesioned and lesioned spinal cord at 14 days post-lesion is shown in cross sections through the spinal cord; asterisks indicate the central canal. **D:** Schematic representation of cell counts in spinal cord sections. All cells are included in the counts, except for those cells that are visible in the last optical section. Scale bar in C = 50 μm.

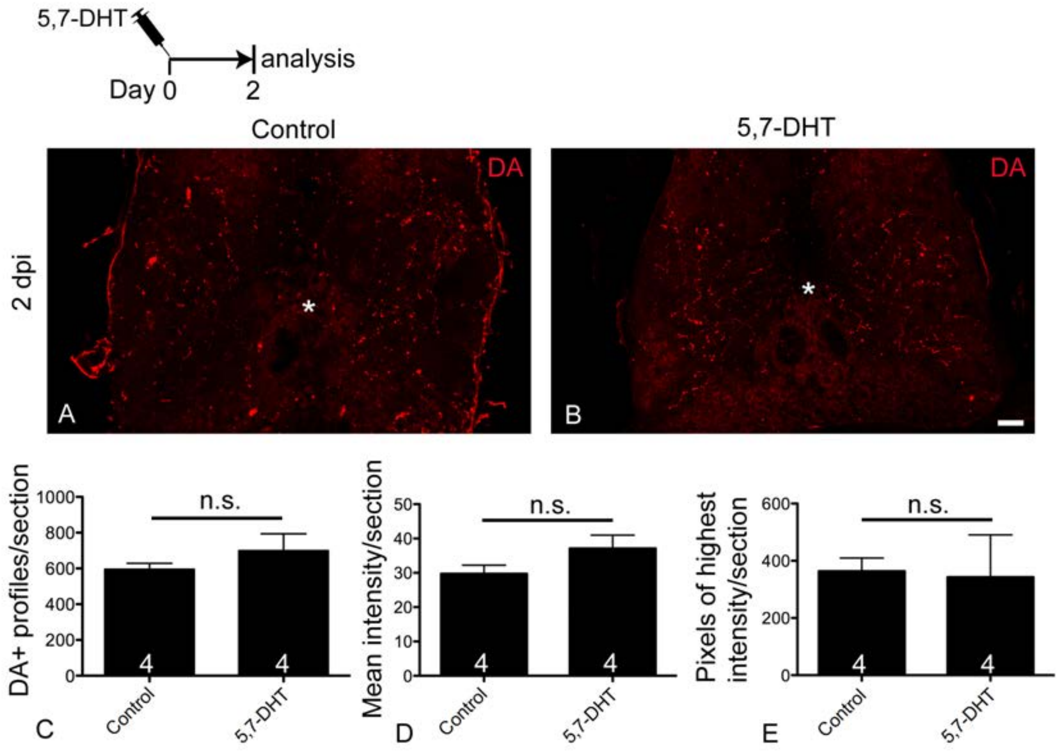

Fig. S3 (related to Fig. 3) Spinal dopamine abundance is not detectably altered after ablation of serotonergic axons with 5,7-DHT. **A,B:** Cross sections through the spinal cord are shown; asterisks indicate the central canal. Immunohistochemistry for dopamine reveals dopaminergic axons in the spinal cord of vehicle-injected and 5,7-DHT injected animals at 2 days post-injection. **C-E:** Number of axonal profiles (Student's t-test, $P = 0.3787$), as well as mean ($P = 0.1875$) and peak ($P = 0.4724$) intensities of dopamine labeling were not detectably different between control and toxin treated animals. Scale bar in B = 50 µm.

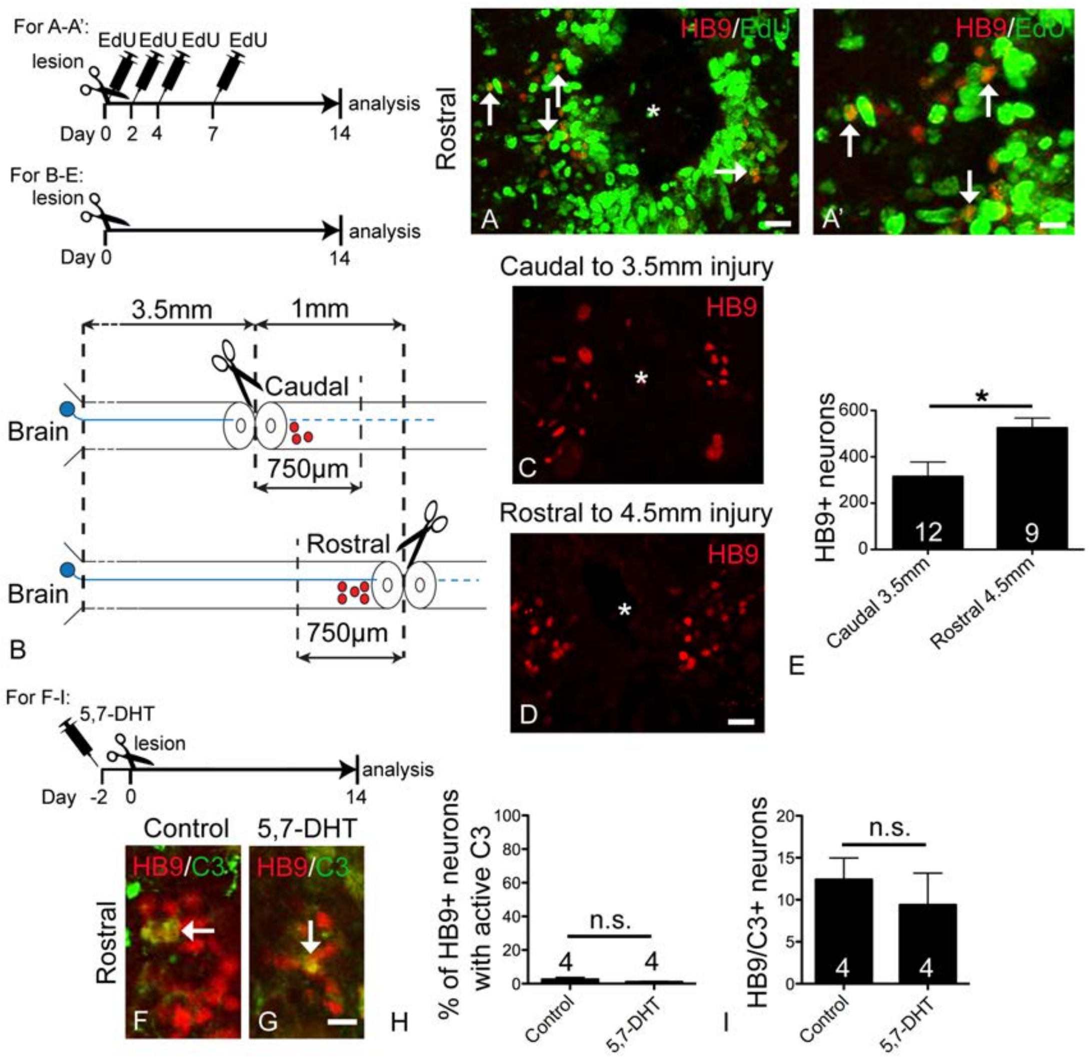

Fig. S4 (related to Fig. 4) Evidence that HB9 immuno-reactive motor neurons are newly generated after a lesion (A, A'), that descending axons influence generation of motor neurons (B-E) and that serotonin signaling does not affect cell death of regenerated motor neurons (F-I). **A,A':** Cross sections through the spinal cord are shown; asterisks indicate the central canal. A' is a higher magnification of the left middle area in A. Multiple EdU injections (see timeline) lead to double labeling of almost half of the newly present HB9$^+$ neurons (arrows) after a lesion. **B-E:** Placing lesions 1 mm apart makes it possible to compare neurogenesis in the same intervening stretch of spinal

cord with either descending axons present (caudal lesion) or absent (rostral lesion) (B). Labeling (C,D) and counts (E) of new $HB9^+$ motor neurons in these equivalent areas up to 750 μm away from the lesion site indicated significantly more new $HB9^+$ motor neurons when the lesion was situated caudal to sampled area (Student's t-test, *P = 0.0184). **F-I:** Double labeling with HB9 and caspase 3 antibodies in control animals and those in which descending serotonergic axons were ablated, did not show increased proportions (Mann-Whitney U-test, P = 0.3429) or numbers (Mann-Whitney U-test, P = 0.6857) of double-labeled cells (arrows) rostral to the lesion site at 14 days post-lesion. Bar in A = 25 μm; in A' = 12.5 μm; in D = 25 μm for C,D; in G = 12.5 μm for F,G.

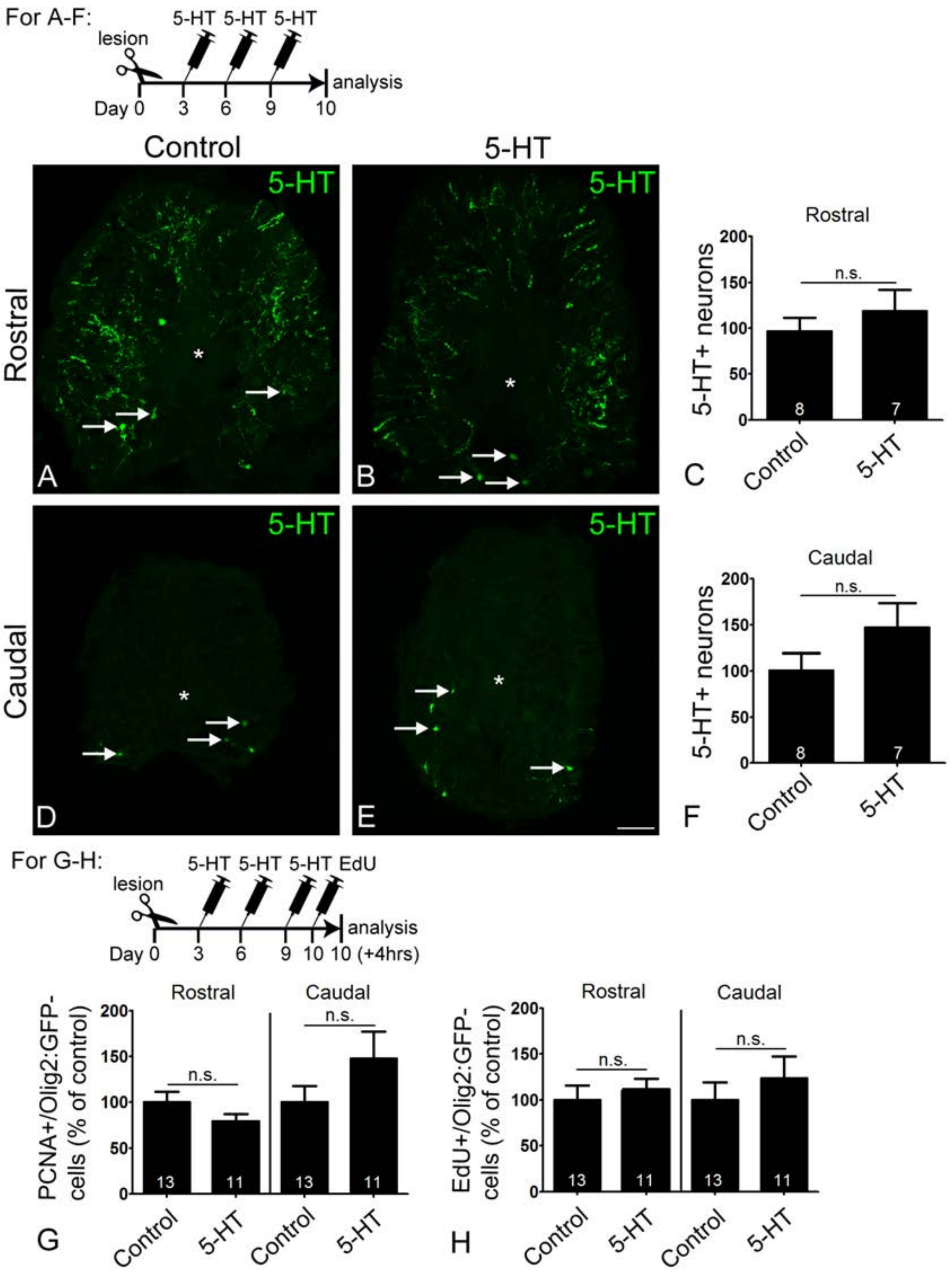

Fig. S5 (related to Fig. 5) Serotonin (5-HT) injections do not increase numbers of serotonergic neurons or proliferation of ventricular cells outside the pMN-like domain. **A-F:** Cross sections through the spinal cord are shown; asterisks indicate the central canal; arrows indicate serotonergic neurons. Serotonin

injections do not significantly increase numbers of serotonergic neurons rostral (Mann-Whitney U-test, $P = 0.5358$) or caudal (Mann-Whitney U-test, $P = 0.2810$) to the lesion site. **G,H:** In contrast to the cells in the olig2:GFP$^+$ pMN-like domain (compare Fig. 4F-I), the number of PCNA$^+$ (Student's t-test, Rostral $P = 0.1640$, Caudal $P = 0.1656$) or EdU$^+$ (Student's t-test, Rostral $P = 0.5525$, Caudal $P = 0.4323$) ventricular cells outside that domain (open arrows in Fig. 4F,G), were not significantly altered by addition of serotonin. Scale bar in E = 50 µm.
